# HIF-1ɑ-regulated miR-1275 maintains stem cell-like phenotypes and promotes the progression of LUAD by simultaneously activating Wnt/β-catenin and Notch signaling

**DOI:** 10.7150/thno.41120

**Published:** 2020-01-22

**Authors:** Neng Jiang, Chang Zou, Ying Zhu, Yifeng Luo, Lili Chen, Yiyan Lei, Kejing Tang, Yu Sun, Wenhui Zhang, Shuhua Li, Qiong He, Jianwen Zhou, Yangshan Chen, Jiping Luo, Wenting Jiang, Zunfu Ke

**Affiliations:** 1 Department of Pathology, The First Affiliated Hospital, Sun Yat-sen University, Guangzhou, Guangdong, China; 2 Clinical Medical Research Center, The First Affiliated Hospital of Southern University, the Second Clinical Medical College of Jinan University, Shenzhen People's Hospital, Shenzhen, Guangdong, China; 3 Department of Radiology, The First Affiliated Hospital, Sun Yat-sen University, Guangzhou, Guangdong, China; 4 Department of Pulmonary and Critical Care Medicine, The First Affiliated Hospital, Sun Yat-sen University, Guangzhou, Guangdong, China; 5 Department of Thoracic Surgery, The First Affiliated Hospital, Sun Yat-sen University, Guangzhou, Guangdong, China.

**Keywords:** MiR-1275, LUAD, Stemness, Wnt/β-catenin, Notch

## Abstract

**Rationale**: Cancer stem cells (CSCs) are considered to be essential for tumorigenesis, recurrence, and metastasis and therefore serve as a biomarker for tumor progression in diverse cancers. Recent studies have illustrated that specific miRNAs exhibit novel therapeutic potential by controlling CSC properties. miR-1275 is upregulated in lung adenocarcinoma (LUAD) and enhances its stemness. However, the underlying mechanisms have not been elucidated.

**Methods**: miRNA expression microarray of LUAD and adjacent nontumor tissues was used to identify miRNAs involved in LUAD malignant progression. miR-1275 expression level was determined using quantitative real-time PCR (RT-qPCR) and in situ hybridization (ISH), and its correlation with clinicopathological characteristics was analyzed in LUAD specimens. The upstream regulator of miR-1275 was validated by chromatin immunoprecipitation (ChIP). The biological functions and underlying mechanisms of miR-1275 were investigated both in vitro and in vivo.

**Results**: MiR-1275 was highly upregulated in lung cancer cell lines and LUAD tissues. Overexpression of miR-1275 in lung cancer patients was associated with shorter overall- and recurrence-free-survival. Proto-oncogene HIF-1ɑ was identified as the transcription mediator of miR-1275. Activation of Wnt/β-catenin and Notch signaling by miR-1275 was found to enhance the stemness of LUAD cells, while antagonizing miR-1275 or suppressing Wnt/β-catenin and Notch pathways potently reversed miR-1275-induced pathway co-activation and stemness. Enhanced stemness dramatically promoted tumorigenicity, recurrence, and metastasis. miR-1275 directly targeted multiple antagonists of Wnt/β-catenin and Notch pathways, including DKK3, SFRP1, GSK3β, RUNX3, and NUMB, respectively, which resulted in signaling activation.

**Conclusions**: Our findings identified miR-1275 as a potential oncogene in LUAD that exerts its tumorigenic effect through co-activating Wnt/β-catenin and Notch signaling pathways. Thus, HIF-1ɑ-regulated miR-1275 might be a potential therapeutic target for LUAD.

## Introduction

Lung cancer is the most common cause of cancer-related deaths globally, with over 40% of cases being lung adenocarcinoma (LUAD) 1. Over the last decade, molecularly targeted therapy using tyrosine kinase inhibitors (TKI) has prolonged the survival of patients with oncogene-driven advanced-stage LUAD 2. However, due to the propensity of LUAD for early dissemination and metastasis, the utility of targeted therapy has reached a plateau, and these challenges represent major obstacles to the improvement of patient survival 3, 4. The major challenge in the management of LUAD patients is the inability to distinguish efficiently between indolent and aggressive tumors. Thus, there is an urgent need to elucidate the mechanisms underlying tumorigenicity and metastasis, which could provide better therapeutic strategies for LUAD.

Tumor progression has been associated with the existence of cancer stem cells (CSCs) within the tumor bulk, which play important roles in metastasis and other malignant phenotypes 5-8. Evolutionarily conserved signaling pathways, including the Wnt/β-catenin, Notch, and Hedgehog, have been shown to be functionally related to stemness control in multiple malignancies 6. The Wnt/β-catenin pathway has been reported to be aberrantly activated in the leukemic stem cells of acute myeloid leukemia (AML) 9, with significant upregulation of the levels of cascade proteins adenomatous polyposis coli (APC) and Axin. Moreover, blocking Wnt/β-catenin signaling with genetic modifications or small-molecule inhibitors has been demonstrated to attenuate cancer stemness 10. Specifically, the ablation of β-catenin leads to a complete regression of CD34+ CSCs in skin tumors. Conversely, the overexpression of β-catenin expands the CSC population 11. Similar to the examples of the Wnt/β-catenin pathway described above, Barnawi et al. found that activation of Notch signaling effectively regulates breast CSCs by inducing the relevant downstream targets predominantly in fascin-positive cells 12. In another study, gene expression profiling in PKCi-silenced glioblastoma CSCs revealed a novel role for Notch signaling in PKCi-mediated glioblastoma CSC survival 13. Notably, studies have shown that Wnt/β-catenin and Notch signaling are simultaneously activated in CSCs 14. However, little is known about their coactivation mechanism.

MicroRNAs (miRNAs), which are a class of endogenous small noncoding RNAs with a length of 21-25 bases, can induce mRNA degradation and suppress gene expression at the posttranscriptional level 15. Various studies have linked aberrant expression and/or the function of miRNAs to tumorigenesis 16. Recently, specific miRNAs have been shown to have promising therapeutic potential by controlling CSCs properties. Ectopic expression of miR-34a 17, miR-145 18 and miR-21 19 is involved in the stemness regulation in several types of cancers, including lung cancer 20. Besides, strategies for increasing or suppressing the levels of miRNAs in tumors by introducing miRNA mimics or antagomiRs have shown promising preliminary clinical results in terms of antitumor effects, particularly if these strategies target CSCs 21.

It has been shown that hypoxia microenvironment ubiquitously exists in a variety of tumor types because of the rapid growth of tumor cells and inefficient supply of blood in the core of the tumor mass 22. Hypoxia microenvironment could lead to hypoxia signaling activation through decreasing degradation of hypoxia-inducible factor 1ɑ (HIF-1ɑ) 23, 24, which is a master transcriptional regulator involved in a series of hypoxia adaptation reactions 25. Numerous studies have revealed that hypoxia regulates the expression of different non-coding RNAs 23, 26. Agrawal R et al. showed that miR-1275 was upregulated in hypoxia microenvironment in glioblastoma 27, suggesting that hypoxia might play a key role in controlling miR-1275 expression. However, the role of HIF-1ɑ in miR-1275 regulation remains poorly understood in LUAD.

Herein, we demonstrated that abnormally expressed miR-1275 could concurrently activate the Wnt/β-catenin and Notch signaling pathways by directly targeting Wnt/β-catenin signaling inhibitors DKK3, SFRP1, GSK3β, and RUNX3 and the Notch signaling antagonist NUMB, thereby enhancing the stemness of LUAD. HIF-1ɑ-regulated miR-1275 was shown to be an important miRNA that is closely associated with tumor progression and poor prognosis, suggesting its clinical significance as a promising prognostic biomarker and potential therapeutic target in LUAD.

## Materials and Methods

### Tissue specimens

Five pairs of primary LUAD tissues and matched adjacent nontumor tissues from the First Affiliated Hospital, Sun Yat-sen University (SYSUFH, Guangzhou, China) were collected to perform miRNA microarrays (Table S1). Additionally, eight pairs of fresh LUAD tissues and matched adjacent nontumor tissues were collected for RT-PCR, and 183 pairs of paraffin-embedded LUAD tissues and matched adjacent nontumor tissues were used for ISH verification. A total of 558 formalin-fixed and paraffin-embedded LUAD tissue specimens were collected from three independent cohorts, including the SYSUFH (n=327), Sun Yat-sen University Cancer Center (SYSUCC) (n=133) and the Central Hospital of Wuhan (CHWH) (n=98), between January 2013 and December 2016. All patients were histopathologically diagnosed by two independent pathologists. The study was approved by the Institutional Research Ethical Committee of SYSUFH with written informed consent from all patients.

### Cell culture

The human bronchial epithelial cell line EBAS-2B and lung cancer cell lines L78, H460, A549, GLC-82, SPC-A1, PC9, H1299, H1975, and H2228 were acquired from the American Type Culture Collection (ATCC, Manassas, VA, USA). A549-luc and H1299-luc cells were obtained from Tang's laboratory (SYSUCC). BEAS-2B cells were maintained in BEGM (Lonza, CC-3170, USA). All lung cancer cell lines were maintained in DMEM or RPMI-1640 (Gibco, Grand Island, NY, USA) supplemented with 10% fetal bovine serum (Gibco, Australia origin, USA) and 1% penicillin-streptomycin (Gibco, Grand Island, NY, USA). Cell lines were validated by short tandem repeat fingerprinting.

### Microarray analysis of miRNAs and mRNAs

MiRNAs were labeled with the miRCURY™ Hy3™/Hy5™ Power labeling kit (Exiqon, Vedbaek, Denmark), and mRNAs were labeled with the Quick Amp Labeling kit (Agilent, USA). Hybridized chips were scanned with an Axon GenePix 4000B scanner (Axon Instruments, Foster City, CA). Differentially expressed miRNAs were determined according to the fold change and *P*-value (fold change≥2, *P*<0.05). The mRNA results were analyzed using GSEA software. The raw miRNA and mRNA microarray data have been deposited into the NCBI Gene Expression Omnibus (GEO) public database (GSE135918 and GSE136043, respectively). The expression levels of the 8 most increased miRNAs in our microarray have been validated. The expression levels of the other differentially overexpressed miRNAs in the microarray are shown in Supplementary Table S2.

### RNA extraction and RT-qPCR

Total RNA was extracted from cultured cells or fresh LUAD tissues with TRIzol reagent (Thermo Fisher Scientific, USA) following the manufacturer's instructions. Quality assessment and quantification of the total RNA were performed using the Nanodrop 2000 spectrophotometer (Thermo Fisher Scientific, USA). Complementary DNA was separately synthesized using a commercial miRNA and mRNA reverse transcription PCR kit (Takara, Dalian, China, 638315 and Takara, Dalian, China, RR047A, respectively). All qPCR analyses were performed using a commercial qPCR reagent kit (TAKARA, RR820A, China) on a real-time PCR detection system (ABI QuantStudio 7, USA). Quantification of mRNAs and miRNAs was normalized to the levels of GAPDH and U6, respectively. All miRNA primers and mRNA primers are listed in Table S3 and Table S4, respectively. The miRNA reverse primers and U6 primers were present in the reagent kit.

### MiR-1275 mimics, inhibitors, small interfering RNAs (siRNAs) and plasmids

MiR-1275 mimics, inhibitors, and siRNAs were chemically synthesized by RiboBio Co. (Guangzhou, China). The Wnt/β-catenin pathway report vector (pGL4.49[luc2P/TCF-LEF RE/Hygro]) and Notch pathway report vector (pGL4[luc2P/RBP-JK RE/Hygro]) were synthesized by Promega (Madison, WI). The sequences of the double-stranded miR-1275 mimics were 5′-GUGGGGGAGAGGCUGUC-3′ and 5′-GACAGCCUCUCCCCCAC-3′. The sequence of the single-stranded miR-1275 inhibitor was 5′-GACAGCCUCUCCCCCAC-3′. Mimics and inhibitors were transfected into the indicated cells using RNAiMax (Life Technologies, USA). siRNAs and plasmids were transfected into the indicated cells using Lipofectamine 3000 (Life Technologies, USA).

### Chemical reagents

The Wnt/β-catenin inhibitor (XAV-939) (S1180, USA) and the Notch inhibitor (RO4929097) (S1575, USA) were purchased from Selleckchem. Cisplatin (CDDP) was purchased from MedChemExpress (Monmouth Junction, NJ, USA). Stock solutions of XAV-939 and RO4929097 were prepared in dimethyl sulfoxide (DMSO) at 1 mM. The stock solution of CDDP was prepared in sterile water at 1mg/ml. XAV-939 and RO4929097 could effectively degrade β-catenin and NICD, respectively, at a concentration of 50.0 µM (24 h). Control cells were treated with the same volume of DMSO.

### In situ hybridization (ISH)

ISH was performed on 5-μm paraffin-embedded sections according to Exiqon's protocol. The miRCURY LNA™ microRNA ISH Optimization Kit (Exiqon, Denmark, Germany) contained 3 digoxin-labeled probes: one double digoxin-labeled probe each for miR-1275 and the negative control and one single digoxin-labeled probe for U6 snRNA as the positive control. The sequence of the miR-1275 probe was Digoxin-5-ACAGCCTCTCCCCCAC-3-Digoxin. The sequence of the U6 probe was Digoxin-5-CACGAATTTGCGTGTCATCCTT-3. The sequence of the negative control probe was Digoxin-5-GTGTAACACGTCTATACGCCCA-3-Digoxin.

### CTC enrichment and measurement

Blood samples (1.0 ml) obtained from patients and were processed within 4 h. For NSG mice, blood samples (0.5 ml) were collected through intracardiac puncture at the experimental endpoints. CTC isolation and enrichment were performed using the NanoVelcro CTC specimen system, as previously described 28. The captured cells were stained with 4', 6-diamidino-2-phenylindole (DAPI, nuclear marker) (CST, 4083S, 1:1000, USA), anti-CD45 antibody (WBC marker) (Abcam, ab64100, 1:400, USA) and anti-CK antibody (cancer cell marker) (Abcam, ab9377, 1:100, USA).

### Immunohistochemistry and immunofluorescence

Immunohistochemistry was performed according to the manufacturer's protocol. Paraffin-embedded slides were incubated with β-catenin (CST, 8480S, 1:100, USA), NICD (Abcam, ab8925, 1:200, USA), SFRP1 (Abcam, ab92552, 1:100, USA), DKK3 (Abcam, ab2459, 1:150, USA), GSK3β (Abcam, ab32391, 1:200, USA), RUNX3 (Abcam, ab92336, 1:150, USA), NUMB (Abcam, ab4147, 1:200, USA), CD133 (CST, 64326S, 1:300, USA), and ALDH1 (Abcam, ab52492, 1:75, USA). Primary antibodies were detected with avidin-biotin-peroxidase complexes with DAB substrate solution (Gene Tech, China).

For immunofluorescence, sections were incubated with β-catenin (CST, 8480, 1:100, USA), NICD antibody (Abcam, ab8925, 1:100, USA), CD133 (CST, 64326, 1:200, USA) and ALDH1 (Abcam, ab52492, 1:200, USA). The nucleus was counterstained with DAPI (CST, 4083, 1:1000, USA). The results were analyzed using a BX63 microscope (Olympus, Tokyo, Japan).

### Flow cytometry

Lung cancer cells were harvested with trypsin and resuspended at a density of 1 × 10^6^ cells/ml in prewarmed DMEM supplemented with 2% FBS and subsequently incubated with or without 100 µM verapamil (Sigma, V4629, USA) for 30 min at 37°C in a water bath. Subsequently, the cells were incubated in a 37°C water bath for 90 min with 5 µg/ml Hoechst 33342 (Sigma, B2261, USA). FACS tubes were shaken every 10 min during incubation. After Hoechst staining, cells were washed twice with ice-cold phosphate-buffered saline (PBS) and resuspended in cold PBS to a final concentration of 1 × 10^6^ cells/ml. Hoechst-positive cells were detected with a flow cytometer (BD Influx) at an excitation wavelength of 350 nm. SP cells were analyzed by FlowJo 7.61 software.

### Western blotting and nuclear and cytoplasmic fraction analysis

All cells and fresh tissues were lysed in radioimmunoprecipitation (RIPA) buffer (Thermo Fisher, USA) supplemented with 1% proteinase inhibitor. We performed nuclear and cytoplasmic fraction analyses according to a standard protocol (Thermo Fisher Scientific, USA). Equal quantities of protein were loaded on a 10% polyacrylamide gel. Proteins were electrophoretically transferred to a polyvinylidene fluoride (PVDF) membrane (Millipore, USA). The PVDF membranes were incubated with primary antibodies at 4°C and secondary antibodies at room temperature. Signals were detected using an ECL chemiluminescence detection system (GE Healthcare Life Sciences and Millipore). The following antibodies were used: β-catenin (CST, 8480S, 1:1000, USA), NICD (CST, ab8925, 1:1000, USA), anti-SFRP1 (Abcam, ab4193, 1:1000, USA), anti-DKK3 (Abcam, ab2459, 1:2000, USA), anti-GSK3β (Abcam, ab32391, 1:2000, USA), anti-RUNX3 (CST, 9647, 1:1000, USA), NUMB (Abcam, ab4147, 1:800, USA), anti-GAPDH (CST, 8884, 1:1000), anti-HISTH3 (ABclonal, A2348, 1:1000), goat anti-mouse IgG-horseradish peroxidase (CST, 7076S, 1:3000, USA), goat anti-rabbit IgG-horseradish peroxidase (CST, 7074S, 1:3000, USA) and peroxidase-conjugated affinity-purified bovine anti-goat IgG (Jackson ImmunoResearch, 805-035-180, 1:5000, USA).

### Luciferase reporter assay

To validate the target genes, we first seeded cells in 24-well plates. After 12 h, the dual-luciferase reporter vectors were transfected into LUAD cells using Lipofectamine 3000 (Invitrogen, L3000-015, USA) according to the instructions of the manufacturer. After 48 h, we harvested the cells and measured the luciferase activity using a Dual-Luciferase Reporter Assay Kit (Promega, E1910, USA). Corning 96-well Solid White Flat Bottom Polystyrene TC-treated microplates (3917, USA) were used for bioluminescence detection. Each experiment was performed in triplicate.

### Xenograft assay

Four- to five-week-old female NSG mice were used to establish a subcutaneous xenograft and metastatic lung model. In the subcutaneous xenograft model, 5×10^3^, 5×10^3^, or 5×10^5^ cells were subcutaneously injected into the flanks of NSG mice (n=6 per group). After inoculation, the tumor size was measured every 5 days using a Vernier caliper. The tumor volume (mm^3^) was defined as follows: volume = (length ×width^2^)/2. In the lung metastatic tumor model, 2 × 10^6^ H1299 cells were injected into the tail vein. Five weeks after the injection, mice were anesthetized with a gas mixture of isoflurane and oxygen, and then. 200 μl of D-luciferin (15 mg/ml in normal saline) (Promega, P1043, USA) was injected into the abdominal cavity. After 10 min, the Xenogen IVIS Spectrum System (Caliper Life Sciences, MA, USA) was applied to evaluate the ability of the different cell lines to metastasize to the lung. This study was approved by the Institutional Animal Care and Use Committee of Sun Yat-sen University.

In the zebrafish model, *Fli1:EGFP* transgenic zebrafish embryos were incubated in a 28°C incubator under established light-cycle conditions. SP cells have been described as CSCs in a variety of tumors, including those from the lung. Several studies on cancer cell lines have demonstrated that SP cells have an increased invasive potential compared with the parental cells 29, 30. Flow cytometry was performed to sort SP cells from the collected A549 cells. Subsequently, SP cells were labeled with Dil (Invitrogen, D3911, Carlsbad, CA, USA), a lipophilic fluorescent tracking dye.

Before injection, zebrafish embryos were dechorionated with microinjection needles and anesthetized with 0.04 mg/ml tricaine (Sigma-Aldrich, USA). A total of 500 Dil-labeled A549-luc cells were injected into the perivitelline cavity of a 2-hpf zebrafish embryo with a Leica microinjector (PLI-100A Plus/Leica S6E), and the embryos were cultured in aquarium water containing 0.2 mmol/L phenylthiourea (PTU, Sigma, USA). The invasion and metastasis of cells were evaluated using an automated fluorescence microscope (Leica DMI8, Germany).

### Statistical analysis

All statistical analyses were performed using SPSS20.0 (IBM, Armonk, NY, USA). The paired Student's t-test (two-tailed) was used for analyses of two groups. The Mann-Whitney U-test and Spearman's correlation analyses were applied to analyze the relationship between miR-1275 expression and the clinicopathological parameters of LUAD. The chi-square test was used to analyze the relationship between miR-1275 expression and miR-1275 target genes. Survival curves were plotted per the Kaplan-Meier method and compared with the log-rank test. Univariate and multivariate Cox proportional hazard models were applied to validate whether miR-1275 was an independent prognostic factor for OS. A nomogram was explored graphically using R software. Receiver operating characteristic (ROC) curves were produced using MedCalc software. The numbers of asterisks indicate statistical significance (* *P* < 0.05; ** *P* < 0.01; *** *P* < 0.001). The data are reported as the mean ± S.D.

## Results

### MiR-1275 is significantly overexpressed in LUAD and associated with tumor progression and poor prognosis

**Figure 1:**
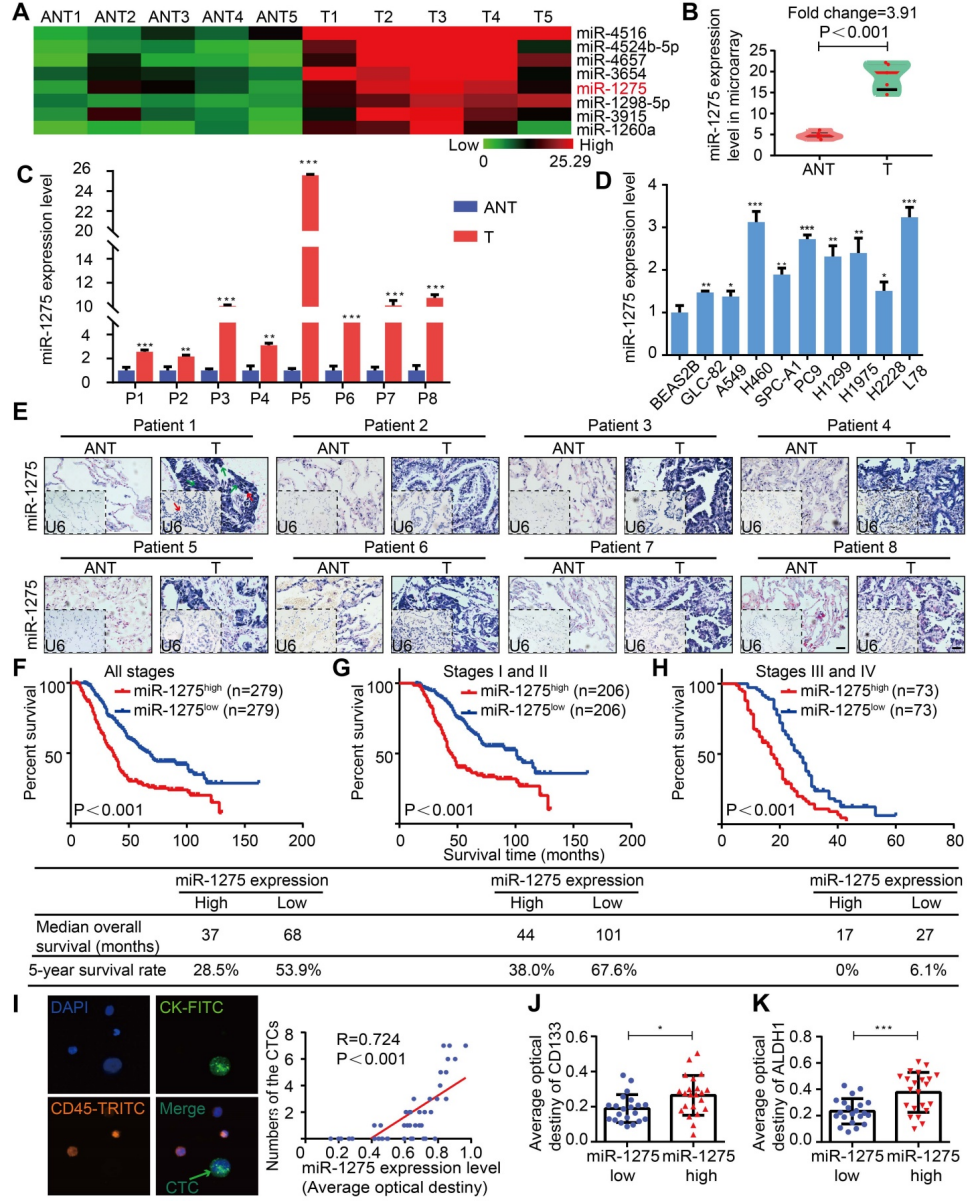
** MiR-1275 is overexpressed in LUAD and correlates with tumor progression and poor survival in LUAD patients.** (A) Heat map showing 8 top upregulated miRNAs in the microarray (fold change ≥2, *P*<0.05). (B) MiR-1275 expression level in the miRNA microarray. (C**)** RT-qPCR analysis of miR-1275 expression in 8 pairs of LUAD tissues and adjacent nontumor tissues. (D) Expression of miR-1275 in normal lung epithelial cell line (BEAS2B) and lung cancer cell lines (n=9). (E) Representative images of ISH showing miR-1275 expression level in tumor tissues and adjacent nontumor tissues. U6, predominately localized in the nucleus, was used as the internal control. miR-1275 is abundantly localized in the membrane, cytoplasm, and nucleus of the tumor cells. The red arrows indicate the nuclei, and the green arrows indicate the cytoplasm and membranes. Scale bar, 50 μm. (F-H) Kaplan-Meier survival analysis of the correlation between miR-1275 expression and the OS of patients in all disease stages (stages I-IV) (F), early stages (stages I-II) (G) and the advanced stages (stages III-IV) (H). (I) Representative images of CTCs in LUAD patients (n=43); CTC numbers were positively correlated with miR-1275 expression. (J-K) Relative CD133 (J) and ALDH1 (K) expression levels in the miR-1275^low^ and miR-1275^high^ expression groups, respectively. Each experiment was performed three times. Data are shown as the mean ± SD, * P < 0.05, ** P < 0.01, *** P < 0.001. ANT: adjacent nontumor tissues, T: tumor.

Tissues were microdissected from 5 pairs of primary LUAD and adjacent nontumor tissues (ANT) and used for RNA extraction followed by miRNA microarray analysis. In this screen, among 8 top overexpressed miRNAs in the microarray, miR-1275 was differentially overexpressed, with consistent and significant upregulation in LUAD tissues compared with their corresponding adjacent nontumor tissues (Figure 1A-C and Figure S1A-B). This finding was further validated by additional data indicating that miR-1275 was ubiquitously overexpressed in a panel of 9 NSCLC cell lines and 183 LUAD samples (150/183) compared to normal lung epithelial cells (BEAS2B) and paired ANT, respectively, as shown in Figure 1D-E and Figure S1C. Interestingly, in 558 LUAD samples from three independent cohorts, the miR-1275 expression levels gradually increased as the tumor stage increased from I to IV (Figures S2A-B). Furthermore, miR-1275 levels were significantly correlated with differentiation degree (*P*<0.001), N classification* (P*<0.001), distant metastasis (*P*<0.001) and clinical stage (*P*<0.001) (Table S5). However, correlation analysis showed that there was no statistical significance in the associations between the miR-1275 level and EGFR^mut^ (*P*=0.185), KRAS^mut^ (*P*=0.725), or ALK^fus^ (*P*=0.512) (Table S6).

Subsequently, to assess the prognostic potential of miR-1275 in LUAD, we analyzed the association between the miR-1275 expression level and overall patient survival. The Kaplan-Meier curves of patients stratified into high and low miR-1275 levels revealed the significantly poorer OS of LUAD patients with high miR-1275 expression levels compared with that of patients with low miR-1275 levels (log-rank test, *P*<0.001, Figure 1F-H). Similar findings were also obtained from SYSUFH, SYSUCC, and CHWH cohorts (log-rank test, *P<0.001*, Figure S3A-I). Furthermore, recurrence-free survival (RFS) was analyzed in patients with recurrence data in SYSUFH, SYSUCC, and CHWH cohorts. As shown in Figure S4, LUAD patients with high levels of miR-1275 exhibited higher tumor recurrence rates than those with low miR-1275 expression levels. The univariate and multivariate analyses showed that miR-1275 expression was an independent predictor of OS (Tables S7-S10).

Additionally, the miR-1275 expression level was positively correlated with an important metastasis-related clinical parameter, the circulating tumor cell (CTC) number (*P*<0.001, R=0.724) (Figure 1I and Table S11). The expression levels of CD133 and ALDH1, markers of CSCs 31, were significantly upregulated in the miR-1275^high^ group (Figure 1J-K and Figures S5A-B). Thus, we speculate that miR-1275 overexpression may promote LUAD progression and distant metastasis by enhancing the stemness, leading to poor prognosis.

### HIF-1ɑ-mediated miR-1275 overexpression enhances the stemness of LUAD cells

**Figure 2:**
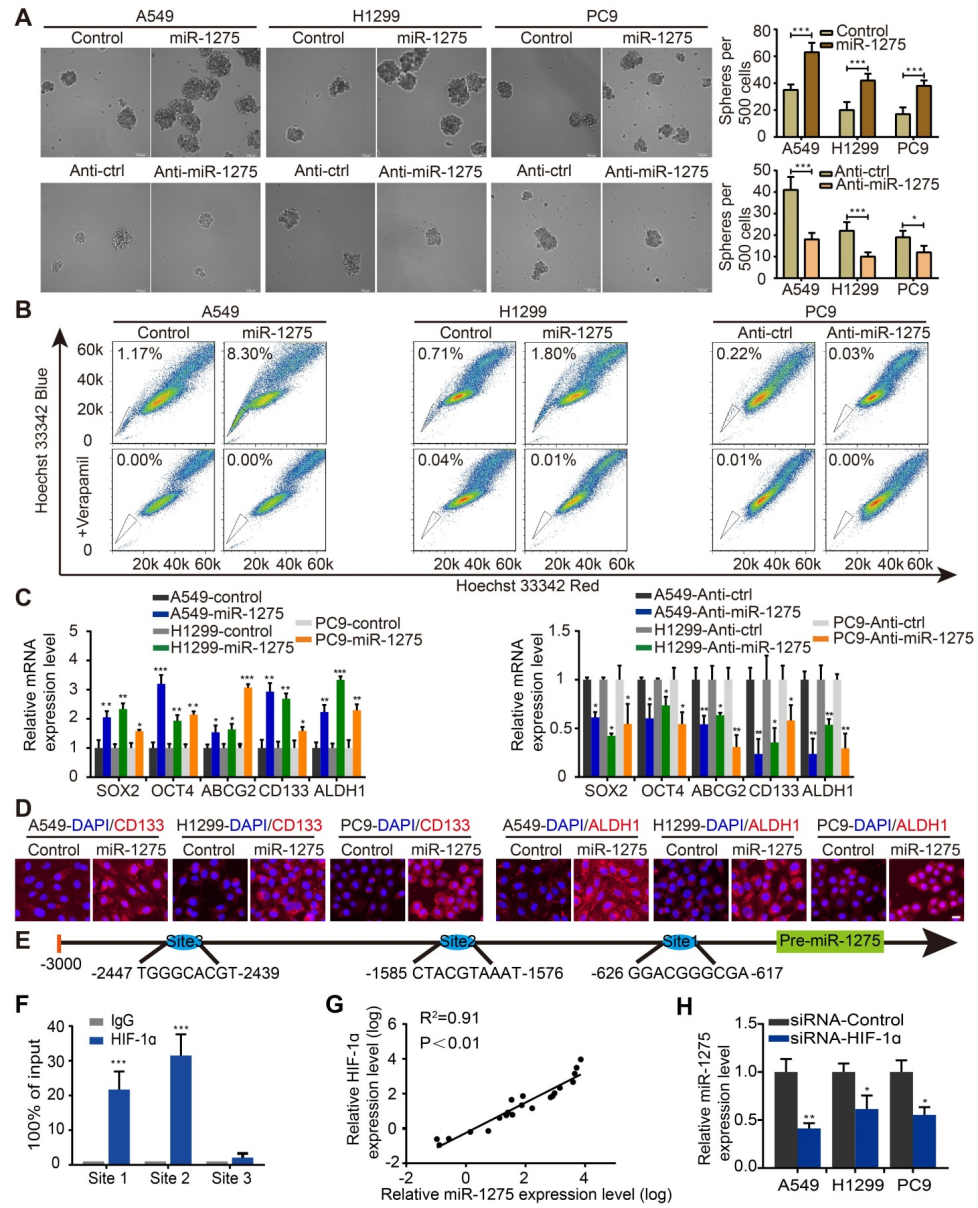
** HIF-1a-mediated miR-1275 maintains stemness in vitro.** (A) Sphere formation ability of stably transfected A549, H1299, and PC9 cells was examined (left panel) and quantified (right panel). Scale bar, 100 μm. (B) Overexpression of miR-1275 results in higher SP cell proportions, whereas the silencing of miR-1275 results in lower SP cell proportions. (C) RT-qPCR analysis for stemness-associated markers, including SOX2, OCT4, ABCG2, CD133, and ALDH1. (D) Subcellular localization of CD133 and ALDH1 was examined by immunofluorescence staining in the indicated cell lines. Magnification, ×200. Scale bar, 50 μm. (E) Predicted binding sites of HIF-1 on the miR-1275 promoter region (site 1, site 2, site 3). (F) Confirmation of HIF-1ɑ binding to miR-1275 promoter region by ChIP analysis in the indicated cells. (G) Pearson's correlation scatter plot of HIF-1α and miR-1275 expression in 20 LUAD clinical specimens. (H) RT-qPCR analysis showing HIF-1α knockdown by siRNA downregulates the levels of miR-1275. Each experiment was performed three times. Data are shown as the mean ± SD, * P < 0.05, ** P < 0.01, *** P < 0.001.

We next explored whether the expression of miR-1275 was related to the stemness of lung cancer cells in vitro. Sphere formation assay was used to investigate the effects of miR-1275 on CSC-like properties. Stably transfected cell lines were constructed by lentivirus transfection (Figure S6). Compared with its expression levels in the corresponding parental cells, the expression levels of miR-1275 in sphere-derived cells were significantly upregulated (Figures S7A-B); the number of spheres increased by approximately 40-50% in the miR-1275-upregulated LUAD cells but decreased in miR-1275-silenced LUAD cells (Figure 2A). Furthermore, miR-1275 inhibitor reversed miR-1275-mediated sphere formation (Figure S7C). Simultaneously, a side population (SP) analysis was performed to detect stem-like cancer cell populations based on their high expression of ABCG2, which is a transporter of Hoechst 33342 and causes its exclusion from cells 32. Consistently, the proportion of side SP cells was markedly increased in miR-1275-overexpressing cells and significantly decreased in miR-1275-silenced cells (Figure 2B and Figure S7D). In addition, the mRNA expression levels of the genes related to CSC-like properties, including SOX2, OCT4, ABCG2, CD133, and ALDH1, were significantly upregulated in the miR-1275-overexpressing LUAD cells and downregulated in the miR-1275-silenced LUAD cells (Figure 2C). The protein expression levels of CD133 and ALDH1 were further verified by immunostaining (Figure 2D). Overall, these data indicate that miR-1275 promotes the CSC-like properties of LUAD cells.

To identify the upstream regulators of abnormal miR-1275 expression in LUAD, its upstream region (-1 to -3,000) was analyzed using UCSC Genome Browser. Three binding sites of hypoxia-inducible factor 1 (HIF-1) were predicted using the intersection of PROMO and JASPAR websites (Figure 2E). Intriguingly, proto-oncogene HIF-1ɑ is a master response subunit of the hypoxic microenvironment, which contributes to cancer progression 26. As shown in Figure 2F, ChIP assay illustrated a high physical binding affinity of endogenous HIF-1ɑ to miR-1275 in the binding sites 1 and 2. Moreover, a positive correlation between miR-1275 and HIF-1ɑ expression level was found in human LUAD specimens as measured by RT-qPCR (Figure 2G), indicating that miR-1275 may be regulated by HIF-1ɑ. To further delineate the regulatory relationship between HIF-1ɑ and miR-1275, HIF-1ɑ was transiently knocked down in indicated cells through siRNA, which led to decreased expression of miR-1275 (Figure 2H). Also, sphere formation could be attenuated after treatment with siRNA-HIF-1ɑ (Figure S7E). Thus, HIF-1ɑ combines with miR-1275 might represent a new therapeutic strategy in LUAD.

### MiR-1275 simultaneously activates Wnt/β-catenin and Notch pathways required for stemness maintenance in vitro

**Figure 3:**
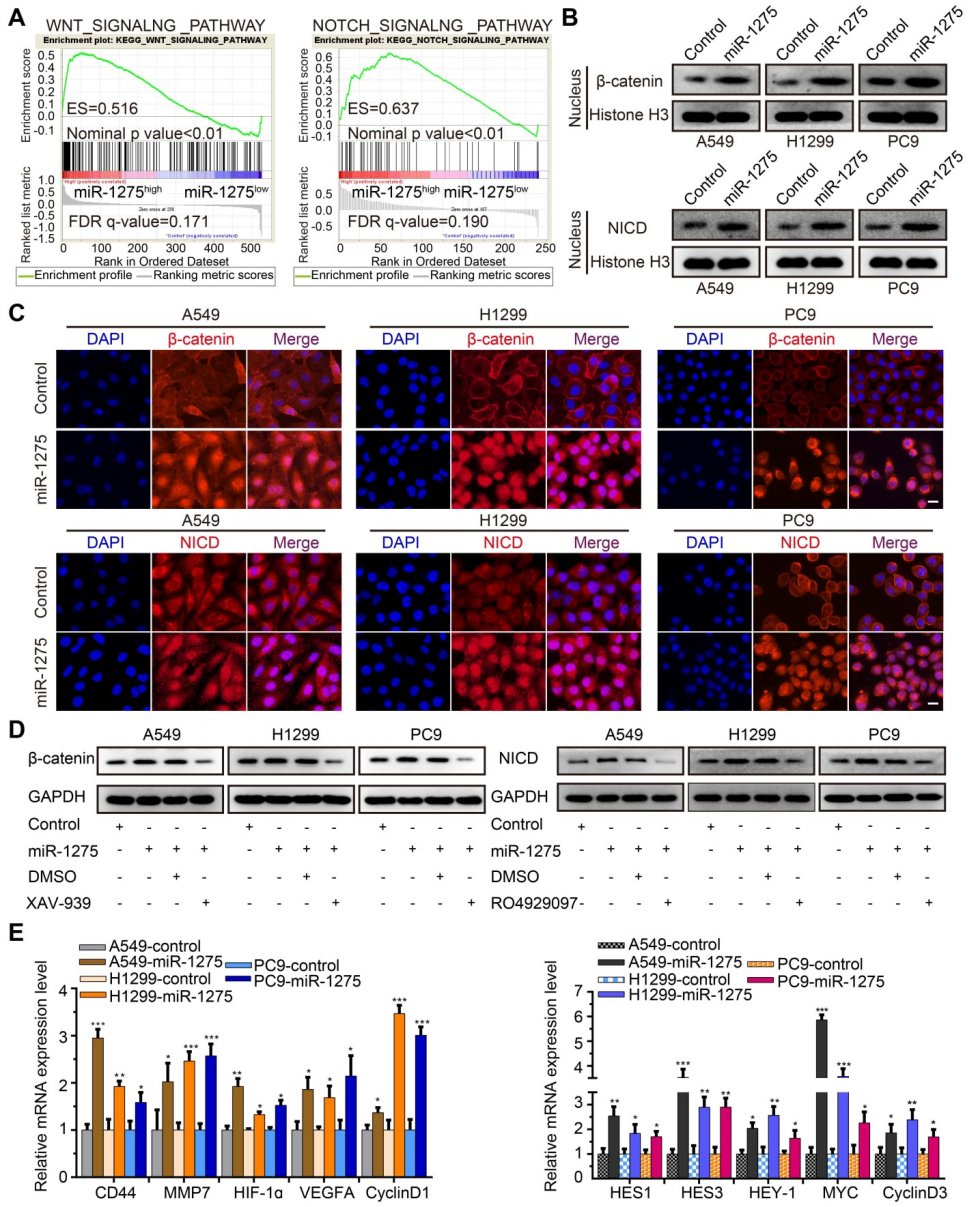
** Overexpression of miR-1275 activates Wnt/β-catenin and Notch pathways.** (A) GSEA analyses indicating enrichment of the Wnt/β-catenin and Notch pathway-related genes in the miR-1275-overexpressing group. (B) Protein levels of nuclear β-catenin and nuclear NICD in the indicated cell lines. (C) Immunofluorescence staining demonstrating cellular locations of β-catenin and NICD in the indicated cell lines. Magnification, ×200. Scale bar, 50 mm. (D) Protein expression levels of β-catenin or NICD in miR-1275-overexpressing cells after treatment with XAV-939 or RO4929097. miR-1275: miR-1275-overexpressing group. DMSO: control group. (E) Relative expression levels of Wnt/β-catenin pathway downstream target genes (CD44, MMP7, HIF-1ɑ, VEGFA, and CyclinD1) and Notch pathway downstream target genes (HES1, HES3, HEY-1, MYC, and CyclinD3) in the indicated cell lines. Each experiment was performed three times. Data are shown as the mean ± SD, * P < 0.05, ** P < 0.01, *** P < 0.001.

Wnt/β-catenin and Notch signaling pathways have been reported to be involved in the progression of NSCLC and are considered crucial for the maintenance of cellular stemness 33. We, therefore, investigated whether miR-1275 simultaneously activated Wnt/β-catenin and Notch signaling. As expected, gene set enrichment analysis (GSEA) showed that miR-1275 overexpression was positively correlated with Wnt- and Notch-activated genes (Figure 3A). Also, compared to the controls, miR-1275 upregulation resulted in increased protein levels of nuclear β-catenin and NICD (Figure 3B-C). Using the pathways reporter assay, miR-1275 overexpression group exhibited increased transcriptional activity of Wnt/β-catenin and Notch pathways (Figure S8A). Rescue assay showed that the protein levels of both β-catenin and NICD displayed a significant decrease or increase when treated with small molecule inhibitor (XAV-939 for Wnt/β-catenin signaling and RO4929097 for Notch signaling, respectively) or miR-1275 mimic (analog of miR-1275), respectively (Figure 3D and Figure S8B). Notably, miR-1275 overexpression upregulated while miR-1275 knockdown downregulated the mRNA levels of downstream genes involved in Wnt/β-catenin and Notch signaling (Figure 3E and Figure S8C). Also, inhibition of Wnt/β-catenin and Notch signaling by small molecule inhibitors caused a significant reduction in the expression of downstream genes (Figure S8D).

Having established that Wnt/β-catenin and Notch signaling are simultaneously activated by miR-1275 in vitro, we next determined the role of their activation in miR-1275-mediated stemness maintenance. In A549, H1299, and PC9 cells, treatment with XAV-939 or RO4929097 led to similar decreases in the levels of miR-1275-mediated sphere formation. When treated with XAV-939 and RO4929097, the sphere formation ability promoted by miR-1275 overexpression decreased to the lowest level (Figure S9A). As shown in Figure S9B, the SP fraction significantly decreased when LUAD cells were cultured in medium supplemented with XAV-939 or RO4929097, and the combined treatment with the inhibitors resulted in the most rapid decrease in the SP fraction. Furthermore, the mRNA expression levels of stemness-related markers, including SOX2, OCT4, ABCG2, CD133, and ALDH1, were decreased to a minimum level in the group treated with the combination of XAV-939 and RO4929097 (Figure S9C). Taken together, our findings indicate that activation of Wnt/β-catenin and Notch signaling is required for miR-1275 to maintain the CSC-like properties in vitro.

### MiR-1275 targets multiple negative regulators of Wnt/β-catenin and Notch signaling

**Figure 4:**
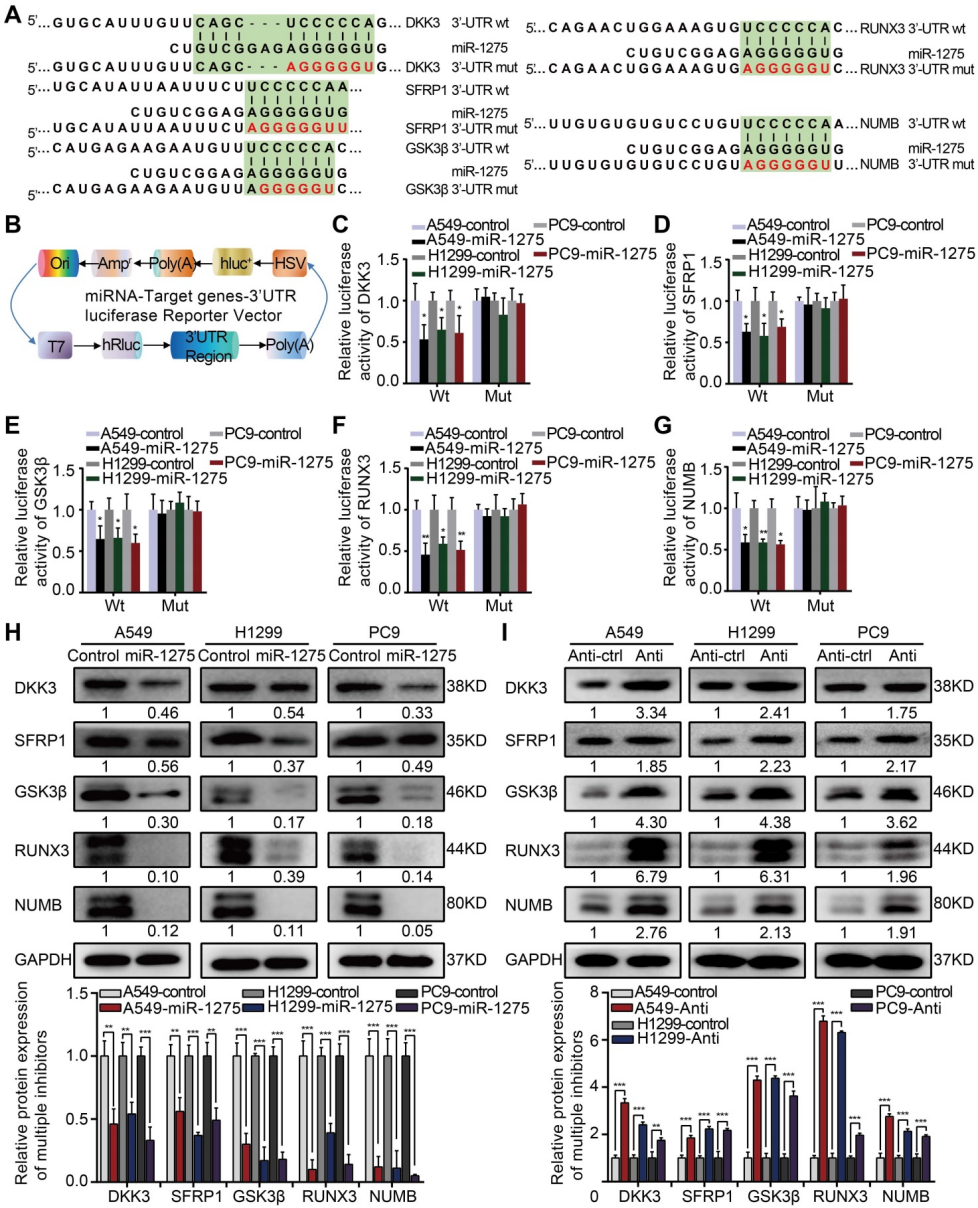
** MiR-1275 directly targets multiple antagonists of the Wnt/β-catenin and Notch signaling pathways.** (A) Schematic of the predicted 3'-UTR wild-type and mutant reporter constructs of the target genes. (B) Construction of the designed dual-luciferase reporter plasmids. (C-G) Relative luciferase activities of the wild-type and mutant reporter plasmids in the A549, H1299, or PC9 cell lines after co-transfection with miR-1275-overexpressing cells or control cells. (H-I) Protein levels of DKK3, SFRP1, GSK3β, RUNX3, and NUMB in the three indicated cell lines were determined by Western blotting. Each experiment was performed three times. Data are shown as the mean ± SD, * P < 0.05, ** P < 0.01, *** P < 0.001.

To identify direct targets of miR-1275, we analyzed miR-1275-binding sites in the 3′-UTRs of transcripts encoding negative regulators of Wnt/β-catenin and Notch signaling using public target prediction tools (TargetScan, miRanda, and Pic Tar). Putative binding sites for miR-1275 were found on genes involved in Wnt/β-catenin signaling, including DKK3, SFRP1, GSK3β, and RUNX3, and Notch signaling, such as NUMB (Figure 4A). Next, we separately cloned the wild-type (WT) and mutant-type (Mut) 3'UTR regions of target genes into the dual-luciferase reporter vector (Figure 4B). The reporter assay indicated that miR-1275 significantly inhibited the luciferase activity of the constructs containing the wild-type 3'-UTR elements of DKK3, SFRP1, GSK3β, RUNX3, and NUMB (Figure 4C-G). Furthermore, marked downregulation in the mRNA and protein levels of DKK3, SFRP1, GSK3β, RUNX3, and NUMB was observed in miR-1275-overexpressing LUAD cells. Conversely, a significant upregulation in the mRNA and protein levels of the same genes was detected in miR-1275-silenced cells. Moreover, miR-1275-knockdown induced upregulation of these antagonistic genes, which could be abolished by treatment with the respective siRNAs (Figure 4H-I and Figure S10A-B). Together, our data indicate that DKK3, SFRP1, GSK3β, RUNX3, and NUMB are direct molecular targets of miR-1275.

### MiR-1275 promotes tumorigenicity, recurrence, and metastasis

**Figure 5:**
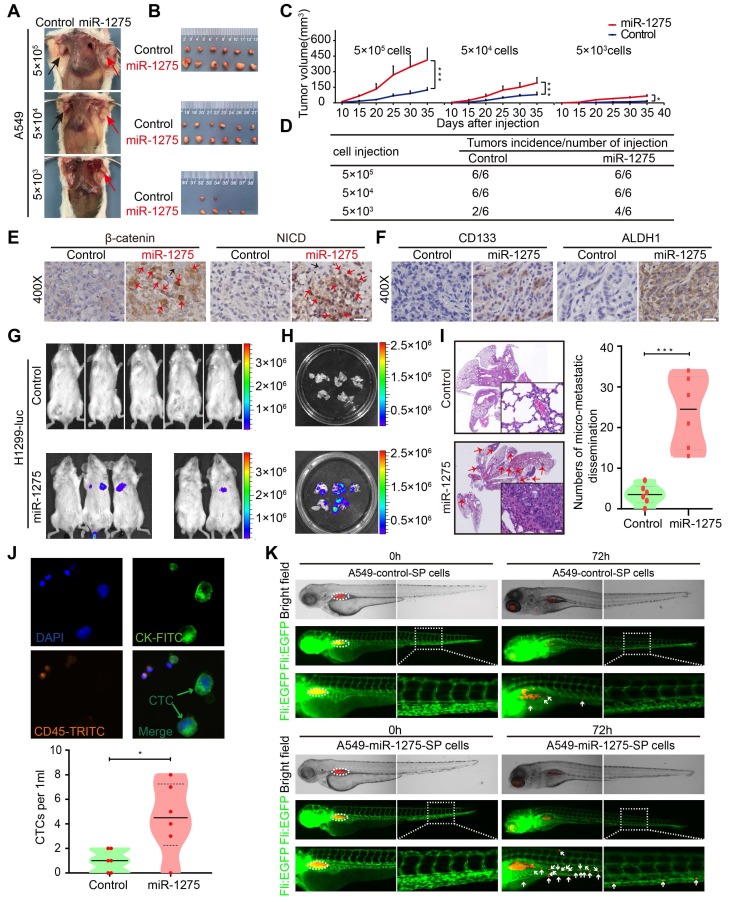
** MiR-1275 promotes tumorigenicity, recurrence, and metastasis by inducing a stem cell-like phenotype.** (A) Effects of miR-1275 on the tumorigenicity of A549 cells at different densities (5×10^5^, 5×10^4^, 5×10^3^). The black arrow indicates the control group (n=6 mice/group), and the red arrow indicates the miR-1275-overexpressing group (n=6 mice/group). (B) Subcutaneous nodules were separated and imaged at the endpoint of the experiment. (C) Tumor growth curves showing growth speed of the tumor nodules. (D) Tumorigenicity rates at different numbers of the indicated cells are shown. (E-F) Immunohistochemistry analyses of β-catenin, NICD, CD133, and ALDH1 in the tumor nodules. Red arrows indicate the nuclear enrichment of β-catenin or NICD. Scale bar, 50 μm. (G) Bioluminescence images of the mouse lung metastasis model. (H) Bioluminescence images of the metastatic lung nodules in the harvested lung tissues. (I) Micro-metastatic lesions were observed and semi-quantified by HE staining. Scale bar, 100 μm. (j) Representative images of captured CTCs intravenously injected in mice. (K) Zebrafishes were photographed at 0 h and 72 h after injection with DiI-red-labeled SP cells. Blood vasculatures are shown in green, implanted tumor areas are indicated by dashed circles, and the disseminated tumor cells are indicated by white arrowheads. Each experiment was performed three times. Data are shown as the mean ± SD, * P < 0.05, ** P < 0.01, *** P < 0.001.

The in vitro assays had exhibited higher cell proliferation ability of miR-1275-overexpressing cells compared with control cells (Figure S11A)- as well as decreased CDDP-induced cell death and better cell viability (Figure S11B). We next examined the effect of miR-1275 on tumorigenicity, recurrence, and metastasis in vivo. In the subcutaneous xenograft model, tumors appeared earlier and larger tumor volumes and higher tumorigenicity rates were observed in miR-1275-overexpressing groups as compared with the control groups (Figure 5A-D and Figure S11C), indicating that miR-1275 might expand the CSC population in LUAD cells. Also, IHC analysis showed higher intranuclear expression of β-catenin and NICD (Figure 5E) and stronger stemness properties marked by the presence of CD133 and ALDH1 in miR-1275^high^ tumors compared to controls (Figure 5F and Figure S11D). Next, we assessed the chemoresistance to CDDP and the relapse ability of LUAD cells with or without miR-1275 overexpression in vivo. As expected, in response to chemotherapy stress induced by CDDP, there was a rapid reduction in the tumor mass. Compared to the slow regrowth of tumors in the control group, CDDP stress-induced reduction of tumor mass continued to regrow rapidly in the miR-1275 overexpressing group (Figure S11E), suggesting that enforced miR-1275 expression desensitized tumor cells to CDDP treatment and induced chemoresistance and recurrence.

In the metastasis model, miR-1275 significantly increased the pulmonary tumor burden as evidenced by the increase in the bioluminescence level (Figure 5G-H), which could be verified by H&E (hematoxylin and eosin) staining (Figure 5I). Furthermore, miR-1275 increased the CTC level in peripheral blood (Figure 5J). The invasion and metastasis ability of A549-miR-1275 SP and A549-control SP cells in zebrafish were examined at 72 h post-injection. The A549-control SP cells resulted in the dissemination of fewer tumor cells from the original tumor sites (upper panel). In contrast, the A549-miR-1275 SP cells induced substantial dissemination of tumor cells to distal regions of the zebrafish body (lower panel) (Figure 5K and Figure S11F), indicating their strong metastatic characteristics. Additionally, we used wound-healing and Transwell invasion assays to validate the effect of miR-1275 on metastasis of LUAD cells in vitro and found a significant increase in the migration and invasion of miR-1275-transfected cells when compared with the control cells (Figure S11G-H). These data demonstrated that miR-1275 might be of potential clinical value not only in monitoring tumorigenicity and recurrence but could also serve as a treatment target for metastatic LUAD.

### Combined predictive role of miR-1275, β-catenin, and NICD in the prognosis and metastasis of LUAD patients

**Figure 6:**
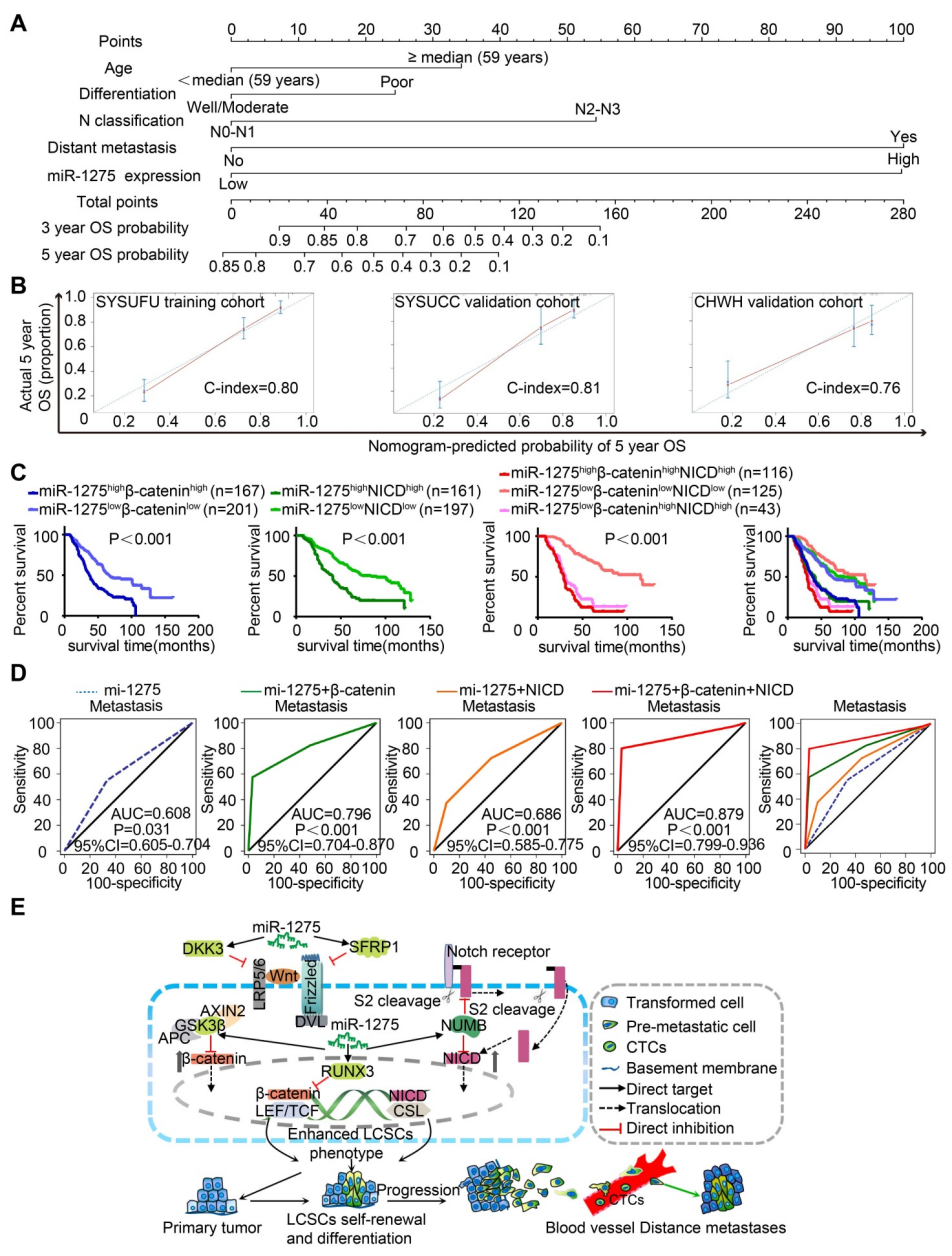
** Combined predictive role of miR-1275, β-catenin, and NICD in the prognosis and metastasis of LUAD patients.** (A) Nomograms were generated according to the multivariate analysis of the SYSUFU cohort to predict 3-year and 5-year OS. (B) Calibration curves indicate the applicability of the nomogram for a 5-year OS. The dashed line indicates the ideal nomogram, and the solid line indicates the actual nomogram. (C) The miR-1275^high^β-catenin^high^NICD^high^ subgroup showed the worst prognosis among the seven subgroups. (D) ROC curves of the prediction models for LUAD metastasis. miR-1275 combined with β-catenin and NICD represented the highest predicted performance for metastasis of the subgroups (AUC=0.879, 95% CI=0.799-0.936, *P* < 0.001). (E) Proposed molecular mechanism model of miR-1275 in this study.

To establish a miR-1275-Wnt/β-catenin- and Notch-signaling-based predictive model, we examined the expression of miR-1275, β-catenin, and NICD in 558 LUAD patients. As shown in Figure S12A-B, the miR-1275^high^ group exhibited higher β-catenin and NICD expression and lower DKK3, SFRP1, GSK3β, RUNX3, and NUMB expression than the miR-1275^low^ group (Table S12 and S13). Based on the multivariate analysis of OS, we constructed a predictive nomogram comprising miR-1275, age, differentiation, N classification, and distant metastasis, which showed more accurate prediction than clinical risk factors for 5-year OS in three independent cohorts (Figure 6A). The calibration plots for the 5-year OS rate were extremely accurate in the three independent cohorts (C-index: 0.80, 0.81, and 0.76, respectively) (Figure 6B). Furthermore, patients with miR-1275^high^β-catenin^high^NICD^high^ displayed the worst median OS (29 months) and the lowest 5-year survival rate (12.5%) compared with the miR-1275^high^β-catenin^high^, miR-1275^high^NICD^high^, and miR-1275^low^β-catenin^high^NICD^high^ subgroups (log-rank test, *P*<0.001). In contrast, patients with miR-1275^low^β-catenin^low^NICD^low^ had the best median OS (115 months) and the highest 5-year survival rate (69.5%) (Figure 6C). Also, using receiver operating characteristic (ROC) analysis, we found that the miR-1275+β-catenin+NICD signature (area under the curve: 0.879; *P*<0.001) had the highest metastasis-predictive function (Figure 6D). As displayed in Figure 6E, ectopic overexpression of miR-1275 suppressed multiple Wnt/β-catenin pathway inhibitors (DKK3, SFRP1, GSK3β, and RUNX3) and a Notch pathway inhibitor (NUMB) resulting in the accumulation of β-catenin and NICD and the induction of Wnt/β-catenin and Notch signaling co-activation. Activated Wnt/β-catenin and Notch signaling further contributed to the stemness, tumorigenicity, and metastasis of LUAD.

## Discussion

Tumor invasion and metastatic disease progression are frequently observed in lung cancer at the initial diagnosis, constituting the leading causes of lung cancer-related deaths 34. Understanding the underlying molecular mechanisms of lung cancer progression is crucial for the development of more effective therapies. NSCLC patients with and without metastasis often exhibit specific miRNA profiles 35. Numerous studies have revealed the association between miRNAs and lung cancer metastasis 36. In this study, we demonstrated that HIF-1ɑ-induced miR-1275 overexpression was significantly associated with metastasis-related parameters in LUAD, including the CTC number, and N and M classifications. The metastasis-promoting properties of miR-1275 have been described in other malignant tumors 37. Interestingly, Kaplan-Meier survival analysis revealed that overexpression of miR-1275 significantly predicts poorer outcomes for LUAD patients than the low expression of miR-1275, indicating that miR-1275 can be used to stratify LUADs into two distinct subgroups with high- and low-risk prognoses. Thus, miR-1275 might help guide individualized follow-up schedules and therapeutic strategies for LUAD patients. To achieve these goals more effectively in the early stages of the disease, it is crucial to characterize the underlying molecular mechanisms of the role of miR-1275 in metastasis.

The presence of CSCs is believed to be a primary cause of tumor metastasis in NSCLC 38. As a small subset of cancer cells within the tumor bulk, CSCs have the ability to self-renew, initiate, and maintain the tumor 39, 40. Emerging studies have shown that miRNAs play important roles in the regulation of stem cell fate and tumor progression in several types of cancers 41, 42, including lung cancer 20. Here, our findings that miR-1275 upregulation enhances sphere formation, promotes stemness, and increases metastasis to the lung point to a pro-metastatic role for miR-1275 in stemness maintenance in LUAD. Here, our study is the first to describe the role of miR-1275 in maintaining the stem cell-like properties of tumor cells, offering a possible molecular mechanism for LUAD metastasis.

A recent study has shown that Wnt/β-catenin and Notch signaling can act in an antagonistic manner to determine cell development and differentiation 43. However, in some cases, Wnt/β-catenin and Notch signaling can be synergistic 44. Our findings revealed that dual activation of Wnt/β-catenin and Notch signaling is at least in part responsible for miR-1275-mediated stemness effects. As negative regulators of Wnt/β-catenin signaling, the loss of DKK3, SFRP1, GSK3β, and RUNX3 might play a major role in NSCLC 45. DKK3, a putative Wnt antagonist, is generally downregulated in human cancers, including lung cancer, and acts as a proapoptotic protein in LUAD cells by decreasing intracellular levels of reactive oxygen species 46. Previous reports demonstrated that SFRP1 inhibits the epithelial-mesenchymal transition in A549 cells 47 and that its promoter is hypermethylated in NSCLC specimens 48.

GSK3β represses the Wnt canonical signaling pathway, and P-GSK3β-ser9 in NSCLC tumors is associated with a short survival time 49. RUNX3 forms a ternary complex with β-catenin/TCF to inhibit Wnt/β-catenin signaling in glioma and gastric, intestinal, and lung cancers 50-52. Overall, these studies suggest that the inhibition or loss of Wnt/β-catenin pathway inhibitors is a prominent feature of NSCLC. Thus, our finding that miR-1275 upregulation contributes to the simultaneous suppression of these genes indicates that targeting of miR-1275 is a therapeutic option in LUAD. More importantly, miR-1275 overexpression has the same inhibitory influence on NUMB as it does on DKK3, SFRP1, GSK3β, and RUNX3. As a Notch pathway suppressor, NUMB inhibits Notch signaling by binding directly to the NICD domain, thus preventing its access to the nucleus 53 or by recruiting ITCH to promote ubiquitination degradation of Notch 54. In NSCLC, expression of NICD1 has been shown to lead to increased proliferative activity, malignant transformation, and tumor growth 55. In the present study, knockdown of miR-1275 led to multilevel inactivation of Wnt/β-catenin and Notch signaling and the subsequent inhibitory effects on CSCs. These findings suggest that miR-1275 is a more effective therapeutic agent for LUAD than previously reported miRNAs, namely, miR-582-3p 20 and miR-708-5p 56.

Our results also provide compelling biological and clinical evidence that miR-1275 serves as a pro-oncogenic microRNA in LUAD. Our findings that overexpression of miR-1275 leads to significant tumor growth, an increased number of CSCs, and enhanced metastasis to lungs point to an in vivo pro-oncogenic role for miR-1275 in LUAD. Ectopic expression of miR-1275 and its oncogenic role in a variety of human cancers, such as lung, bladder, and colorectal cancers, have been illustrated recently 57-59. Specifically, in NSCLC, a recent study discovered that more than 74% (52/70) NSCLC tissues exhibited an up-regulated miR-1275 level when compared with normal lung tissues. High level of miR-1275 can significantly promote NSCLC cell proliferation and metastasis through directly targeting LZTS3 57, indicating its oncogenic function. However, several studies reported that miR-1275 could also serve as a tumor suppressor in gastric cancer and hepatocellular and nasopharyngeal carcinomas 60-62. These observations suggest that miR-1275, depending on the cellular context, serves both as an oncogene and a tumor suppressor. Similar results were also observed with other miRNAs, including miR-582-5p and miR-186 20. Thus, in the case of miR-1275, tumor microenvironment, epigenetic modifications, and immune microenvironment may participate in modulating its multidimensional roles in malignant tumors. Furthermore, due to the close correlation between high miR-1275 and low DKK3, SFRP1, GSK3β, RUNX3, and NUMB expression levels in LUAD specimens and cell lines, aberrant expression of miR-1275 may serve as a novel mechanism for the activation of Wnt/β-catenin and Notch signaling. This conclusion was further reinforced by the in vivo evidence as well as from the multicenter clinical sample data that ectopic miR-1275 overexpression results in increased β-catenin and NICD translocation to the nucleus, leading to the activation of Wnt/β-catenin - and Notch-responsive genes, respectively 41, 63.

Wnt/β-catenin and Notch signaling pathways have a critical role in the maintenance of stem-like properties; this role is well defined in a wide variety of cancer types, providing a new therapeutic strategy to ameliorate the metastasis of malignant tumors 64. For example, Vantictumab (OMP-18R5), a mAb that blocks five Fz receptors (Fz1, Fz2, Fz5, Fz7, and Fz8), is under investigation in phase I studies 65. In addition, the oral GSI PF-0308414 inhibitor has demonstrated promising clinical activity in a phase I dose-finding study in patients with advanced solid tumors 66. However, small-molecule drugs usually act against a single molecule. In contrast, miRNAs function as master regulators of cellular genes and exert strong regulatory effects on tumor development and progression by targeting multiple molecules, making them promising candidates as therapeutic targets (in the form of miRNA mimics or antimiRs) 67. Our current study revealed that miR-1275 had important effects on CSCs by activating Wnt/β-catenin and Notch signaling to restore stemness-related gene expression. In particular, the combined evaluation of the expression of miR-1275, β-catenin, and NICD could further stratify LUAD into two distinct subgroups with high- and low-risk prognoses. A nomogram, including miR-1275, might provide simple and accurate prognostic predictions for LUAD. Therefore, in the biological context of β-catenin and NCID, miR-1275 expression might be helpful clinically during the treatment decision-making process of LUAD patients.

HIF-1ɑ, a master response subunit of the hypoxia microenvironment, is known to mediate the progression of various cancers 26, 68. Previous studies have illustrated that HIF-1ɑ could mediate the expression of several miRNAs in malignant cancers, including miR-224 68, miR-424 69, and miR-382 70. In this study, we uncovered that HIF-1α can directly bind to the upstream promoter of miR-1275 to induce miR-1275 overexpression. This finding enriches our knowledge about the regulatory mechanism of miR-1275 under hypoxia. In summary, HIF-1ɑ-mediated miR-1275 overexpression has the potential to act as a prognostic biomarker in LUAD. Overexpression of miR-1275 accelerates the accumulation of β-catenin and NICD and enhances the stem-like traits of LUAD cells by activating Wnt/β-catenin and Notch signaling, consequently promoting tumorigenesis, recurrence, and metastasis. Our study has not only identified the miR-1275/Wnt/β-catenin and miR-1275/Notch axes as critical regulatory pathways in LUAD CSCs but also provides a novel therapeutic target for the treatment of LUAD.

## Supplementary Material

Supplementary figures and tables 1, 3-13.

Supplementary table 2.

## Abbreviations

MiRNA: MicroRNA
LUAD: Lung adenocarcinoma
LCSCs: Lung cancer stem cells
ISH: In situ hybridization
IHC: Immunohistochemistry
WB: Western blot
SYSUFH: The First Affiliated Hospital of Sun Yat-sen University
SYSUCC: Sun Yat-sen University Cancer Center
CHWH: Central Hospital of Wuhan
AUC: Area under curve
DKK3: Dickkopf WNT signaling pathway inhibitor 3
SFRP1: Secreted frizzled-related protein-1
GSK3β: Glycogen synthase kinase-3β
RUNX3: Runt-related transcription factor 3
NUMB: NUMB endocytic adaptor protein
DLL1: Delta like canonical Notch ligand 1
HIF-1ɑ: hypoxia-inducible factor 1ɑ
GSEA: Gene set enrichment analysis
H3: HISTH3
