# Supplementary material for: HIF-1ɑ-regulated miR-1275 maintains stem cell-like phenotypes and promotes the progression of LUAD by simultaneously activating Wnt/β-catenin and Notch signaling
Source: Theranostics. 2020 Jan 22;10(6):2553–70. doi: 10.7150/thno.41120 (PMC7052895; doi:10.7150/thno.41120)
Supplement: Supplementary file 1 — Supplementary figures and tables 1, 3-13. [file thnov10p2553s1.pdf]

Supplementary Materials

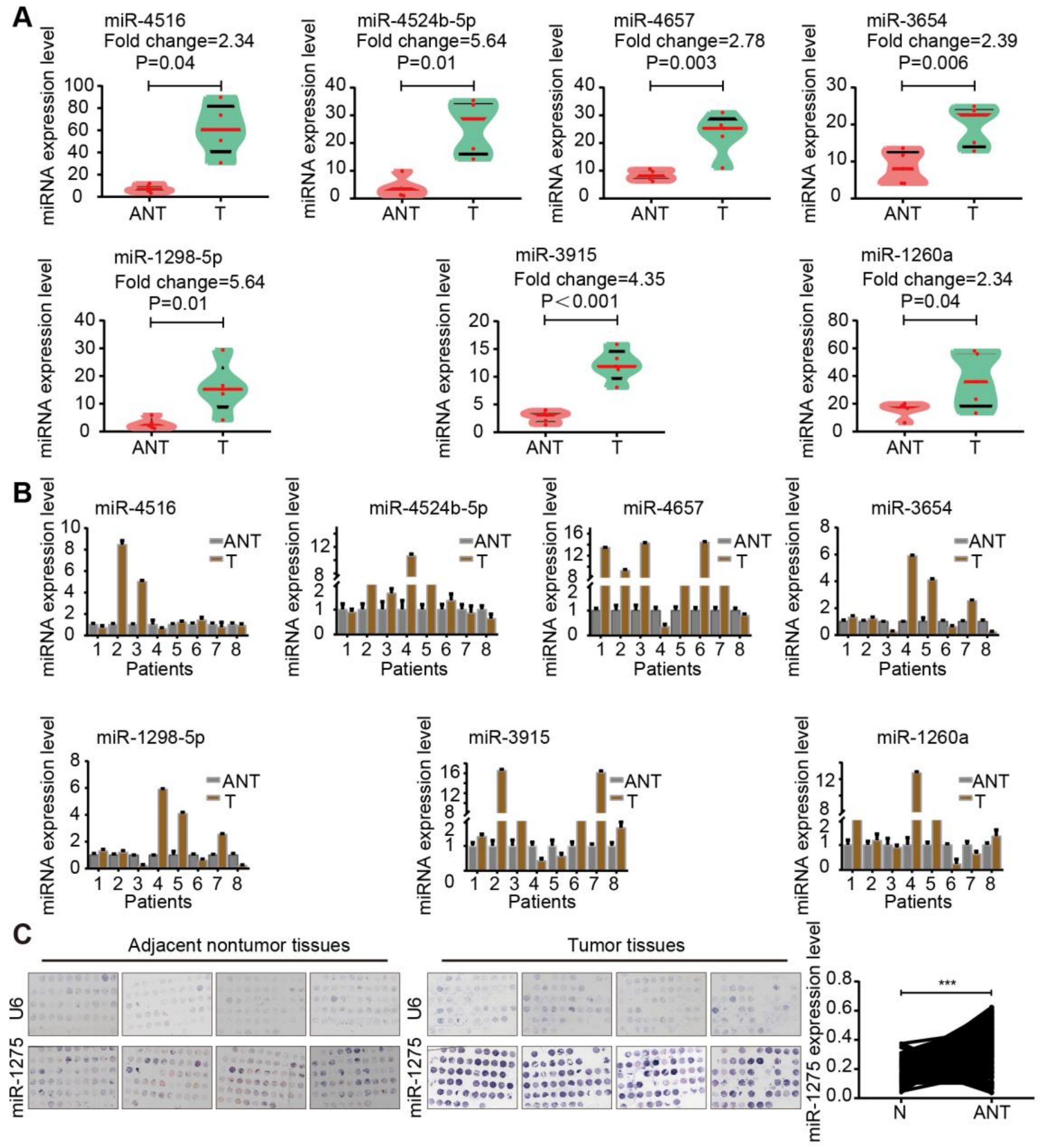

Figure S1: Expression levels of the other top 7 overexpressed miRNAs. (A) Expression levels of miR-4516, miR-4524b-5p, miR-4657, miR-3654, miR-1298-5p, miR-3915, and miR-1260a in the microarray analysis. (B) Expression levels of miR-4516, miR-4524b-5p, miR-4657, miR-3654, miR-1298-5p, miR-3915, and miR-1260a in 8 pairs of LUAD tissues and adjacent nontumor tissues. U6 was used as an internal reference. Each experiment was performed three times. (C) miR-1275

expression level in 183 pairs of paraffin-embedded LUAD tissues and matched adjacent nontumor tissues. ANT: adjacent nontumor tissues, T: tumor.

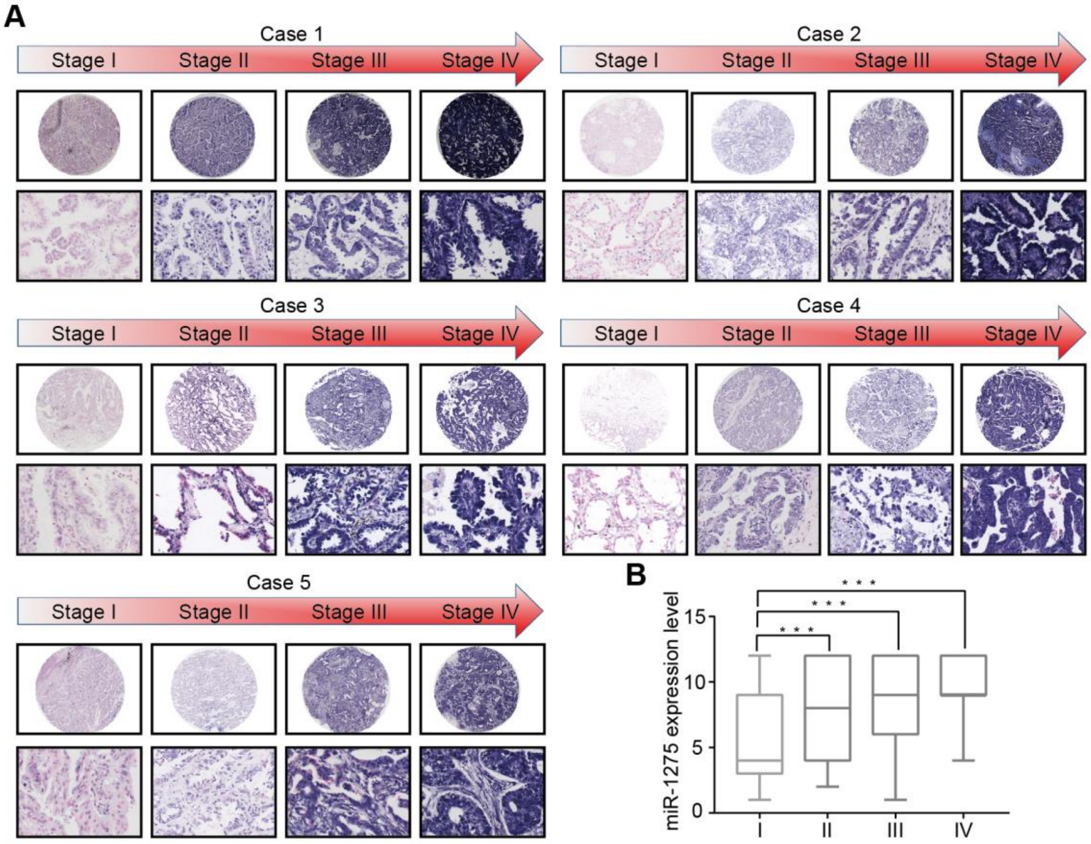

Figure S2: MiR-1275 expression levels at different clinical stages. (A) Representative ISH images. (B) Box-Whisker diagram showing the miR-1275 expression level at each clinical stage (I-IV). Data are reported as the mean ± SD (**P* < 0.05, ***P* < 0.01, ****P* < 0.001).

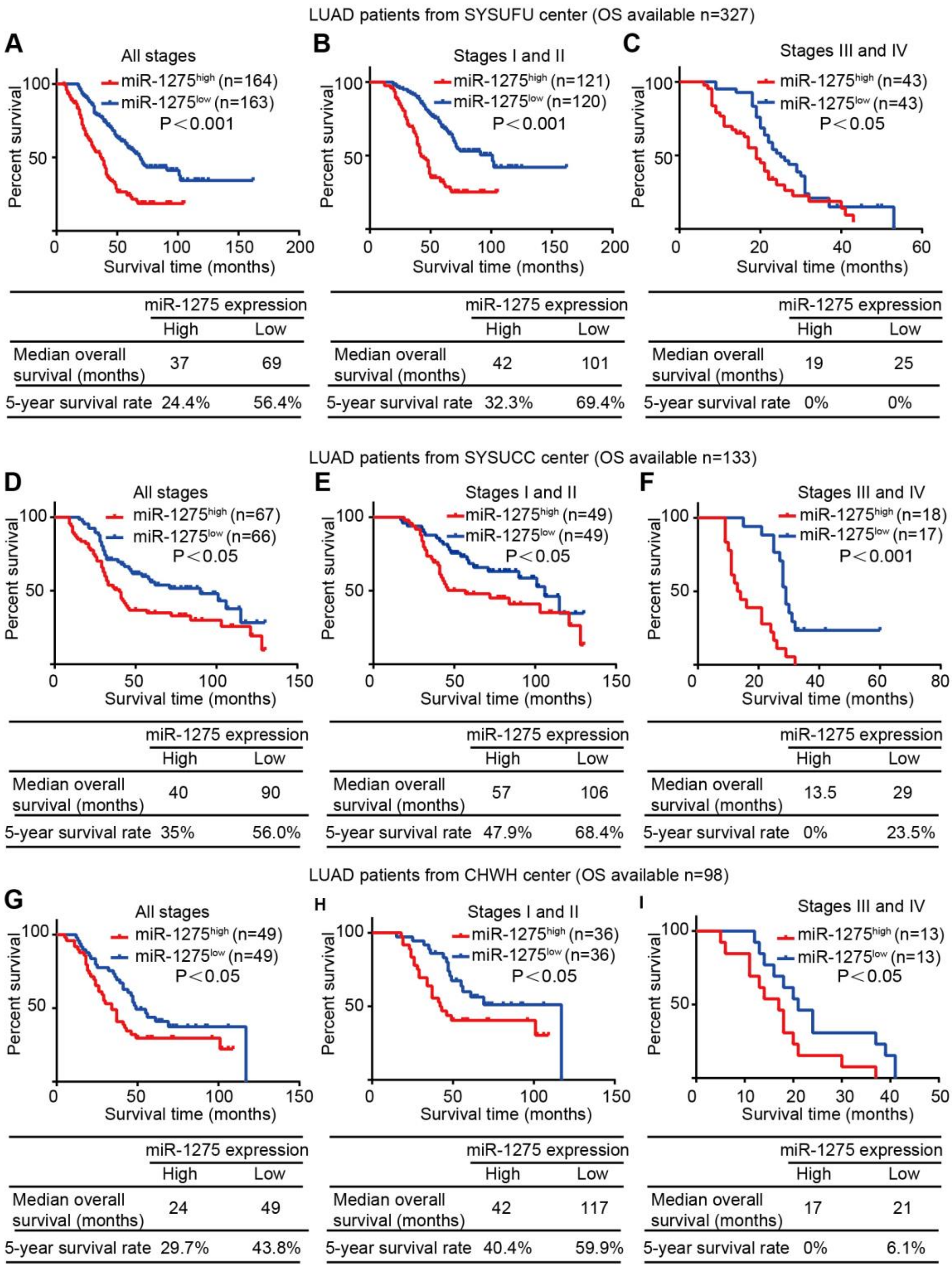

	miR-1275 expression	
	High	Low
Median overall survival (months)	37	69
5-year survival rate	24.4%	56.4%

	miR-1275 expression	
	High	Low
Median overall survival (months)	42	101
5-year survival rate	32.3%	69.4%

	miR-1275 expression	
	High	Low
Median overall survival (months)	19	25
5-year survival rate	0%	0%

	miR-1275 expression	
	High	Low
Median overall survival (months)	40	90
5-year survival rate	35%	56.0%

	miR-1275 expression	
	High	Low
Median overall survival (months)	57	106
5-year survival rate	47.9%	68.4%

	miR-1275 expression	
	High	Low
Median overall survival (months)	13.5	29
5-year survival rate	0%	23.5%

	miR-1275 expression	
	High	Low
Median overall survival (months)	24	49
5-year survival rate	29.7%	43.8%

	miR-1275 expression	
	High	Low
Median overall survival (months)	42	117
5-year survival rate	40.4%	59.9%

	miR-1275 expression	
	High	Low
Median overall survival (months)	17	21
5-year survival rate	0%	6.1%

Figure S3: miR-1275 expression level is positively associated with poor OS in three independent clinical centers. (A-I) Kaplan-Meier analysis for OS and the 5-year survival rates of LUAD patients at all stages (left panel), early stages (central panel), and advanced stages (right panel) from the SYSUFH (A-C), SYSUCC (D-F)

and WUHAN center (G-I). Statistical analysis was performed with the log-rank test (*P < 0.05; **P < 0.01; ***P < 0.001).

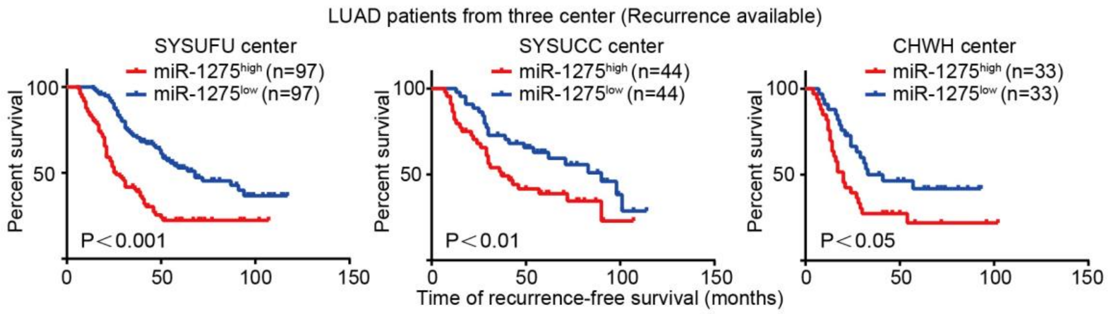

	miR-1275 expression		miR-1275 expression		miR-1275 expression	
	High	Low	High	Low	High	Low
Median RFS time (months)	26	68	26	110	20	30
5-year recurrence rate	87.9%	60.1%	90.2%	64.7%	91.5%	69.3%

Figure S4: Overexpression of miR-1275 is associated with RFS in patients from the SYSUFU center (left panel), SYSUCC center (central panel), and CHWH center (right panel).

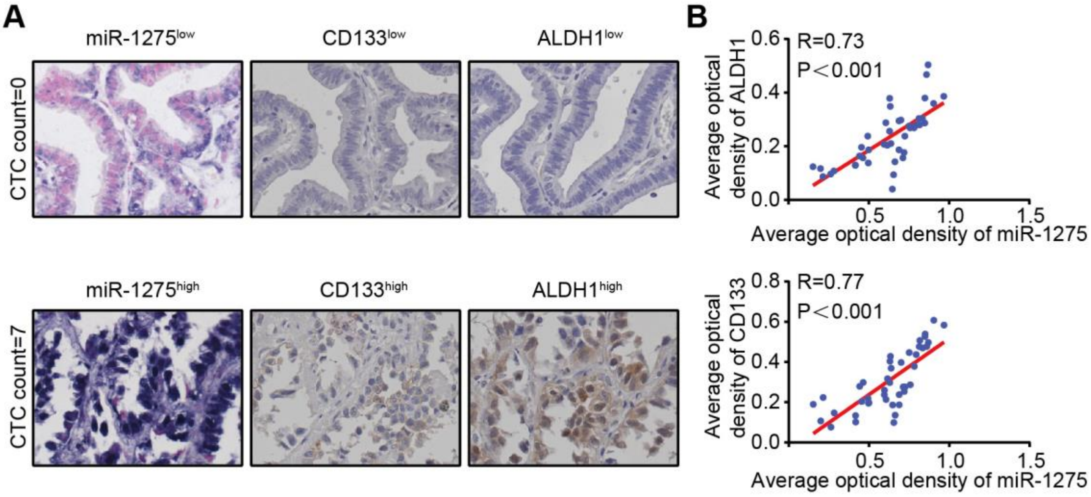

Figure S5: The miR-1275 expression level is positively correlated with the CTC count and CD133 and ALDH1 expression levels. (A) Representative images showing the correlation between the CTC count and the miR-1275, CD133 and ALDH1 levels. Scale bar, 100 μm. (B) Correlation analysis between the miR-1275 level and CD133 or ALDH1 level.

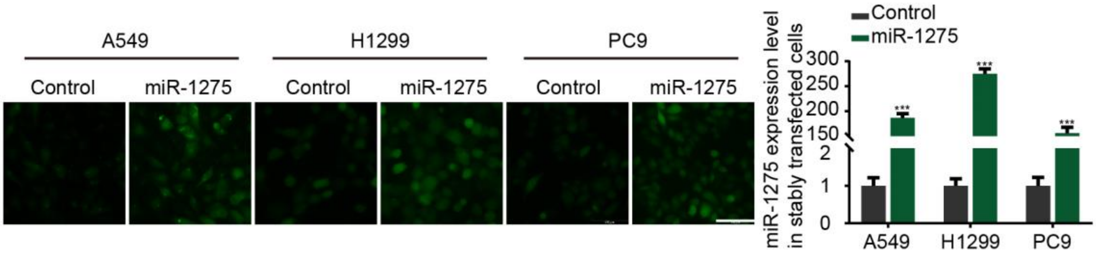

Figure S6: Transfection efficiency of miR-1275 in three cell lines. Representative fluorescence images of A549, H1299, and PC9 cells transduced with GFP-control lentivirus or GFP-miR-1275 lentivirus. Green fluorescent protein (GFP) is indicated in green. Scale bar: 100 μm and ***P < 0.001.

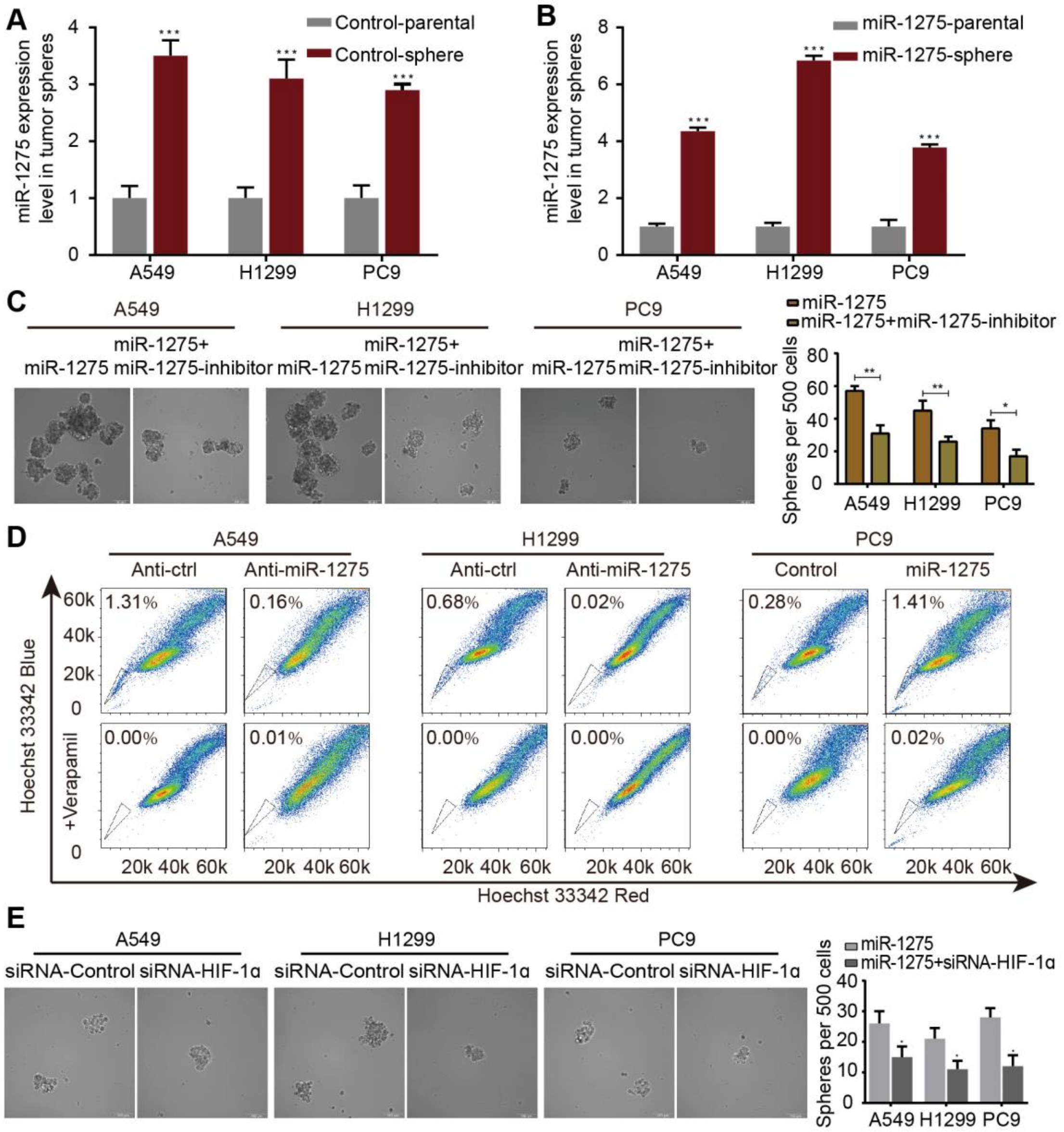

Figure S7: MiR-1275 promotes stem cell-like properties in the indicated cell lines. (A) Relative miR-1275 expression levels in the control-parental cells and control-sphere cells. (B) Relative miR-1275 expression levels in the miR-1275-parental cells and miR-1275-sphere cells. (C) miR-1275-induced sphere formation could be reversed by using a miR-1275 inhibitor. (D) Upregulation of miR-1275 increased the SP cell proportions, whereas knockdown of miR-1275 lowered the SP cell proportions. SP cell proportions were determined by Hoechst 33342 dye exclusion. Anti-ctrl: miR-1275 sponge control. (E). Sphere formation

ability could be attenuated after treatment with siRNA-HIF-1α. (*$P < 0.05$, **$P < 0.01$, ***$P < 0.001$).

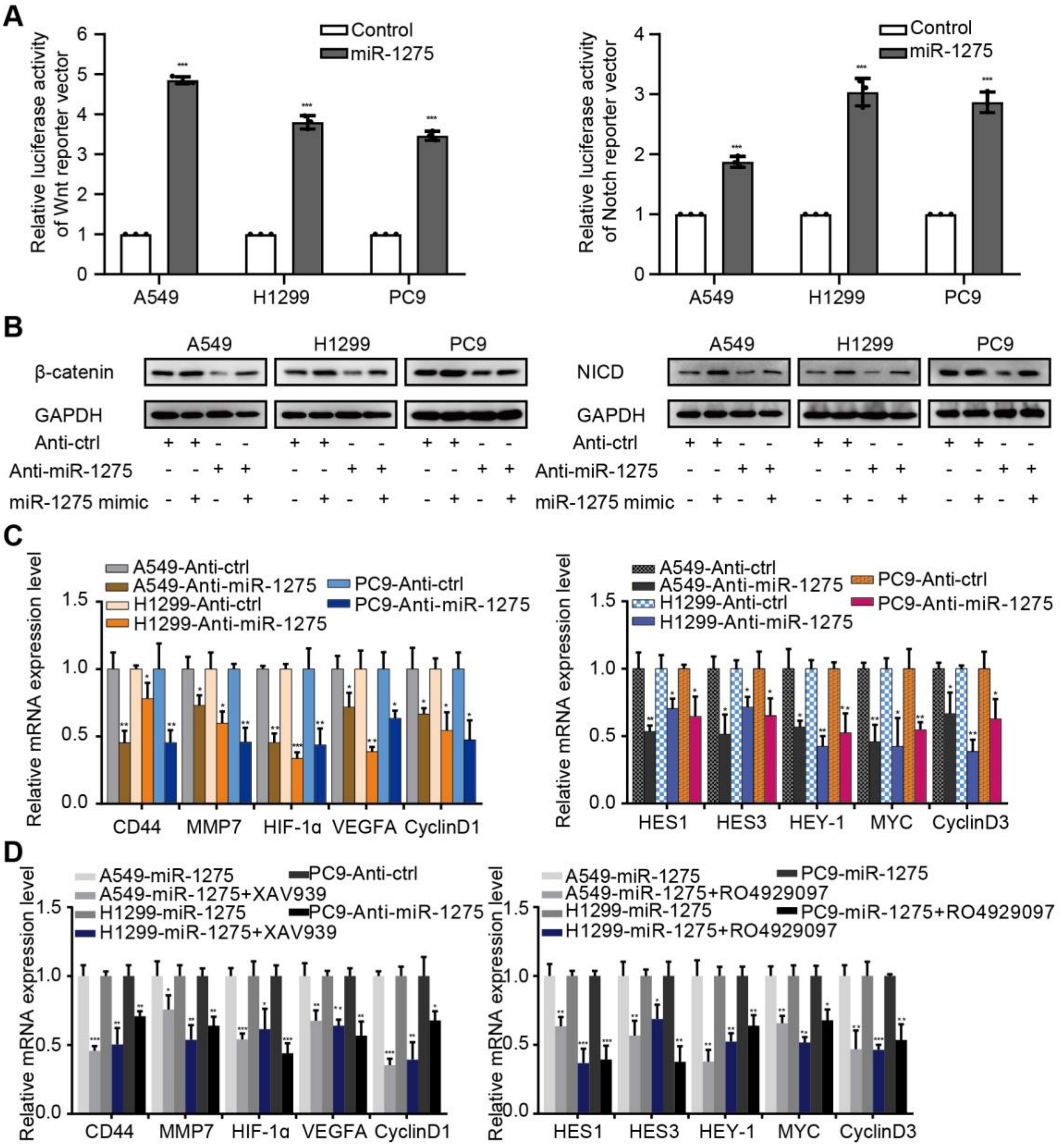

Figure S8: MiR-1275 simultaneously activates the Wnt/β-catenin and Notch signaling pathways. (A) Relative luciferase activity of Wnt/β-catenin (left panel) and Notch (right panel) reporter vectors in the indicated cells. (B) Protein levels of β-catenin and NICD in miR-1275-silencing cells after treatment with miR-1275 mimic, an analog of miR-1275. (C) RT-qPCR analysis of the established downstream target genes of the Wnt/β-catenin or Notch pathway in miR-1275 knockdown cell lines. (D) mRNA expression levels of downstream molecules after treatment with

small molecule inhibitors. *$P < 0.05$, **$P < 0.01$, ***$P < 0.001$.

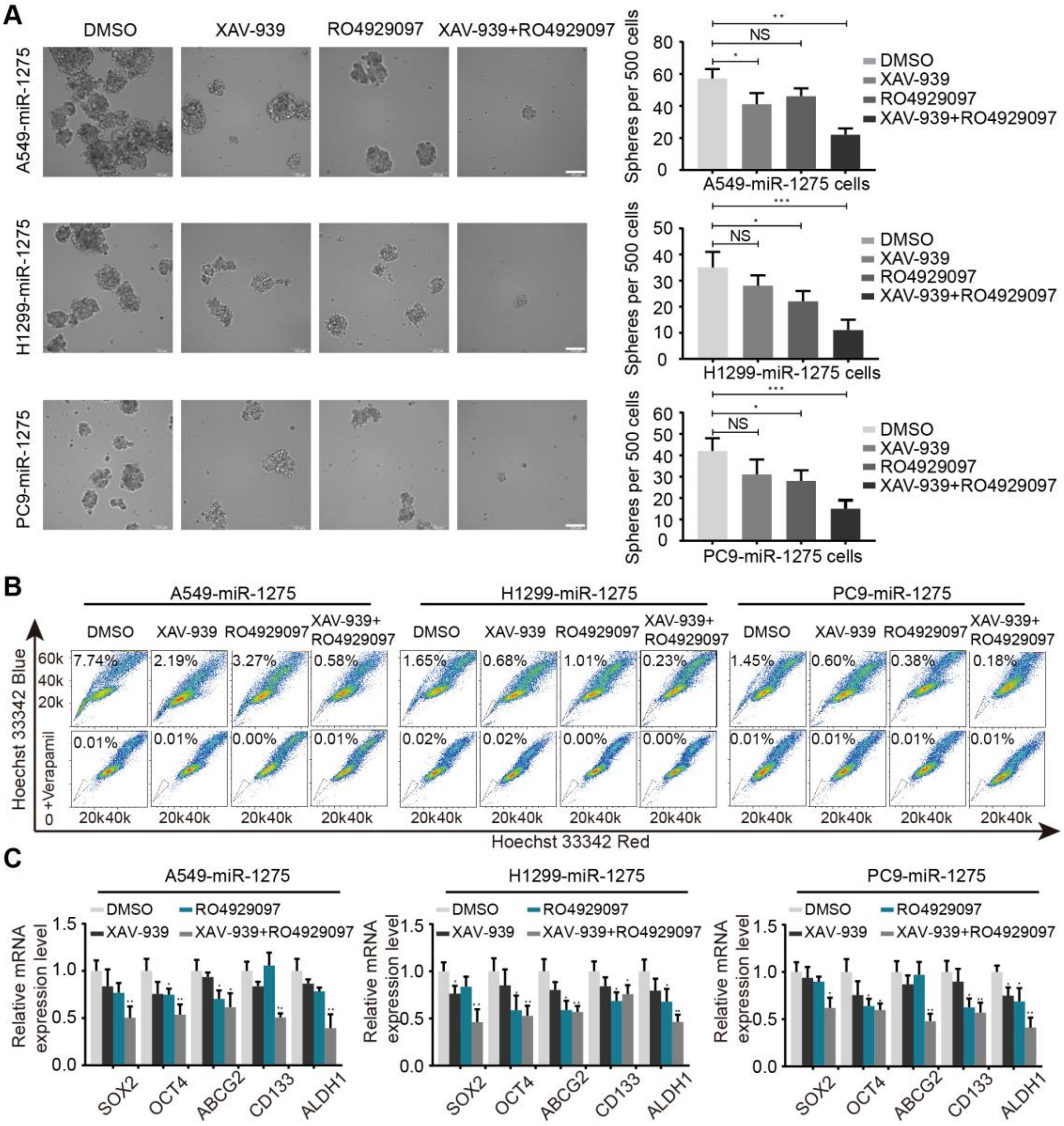

Figure S9: Mir-1275 maintains stemness by simultaneously activating the Wnt/β-catenin and Notch pathways. (A-B) Formation of tumor spheres and proportions of SP cells are shown after treatment with XAV-939, RO4929097 or XAV-939 plus RO4929097 in miR-1275-overexpressing cell lines. (C) Expression levels of stemness-related markers (SOX2, OCT4, ABCG2, CD133, and ALDH1) in

miR-1275-overexpressing cell lines after treatment with XAV-939, RO4929097, or XAV-939 plus RO4929097. DMSO: control. Data are shown as the mean ± SD, * *P* < 0.05, ** *P* < 0.01, *** *P* < 0.001. NS: not statistically significant.

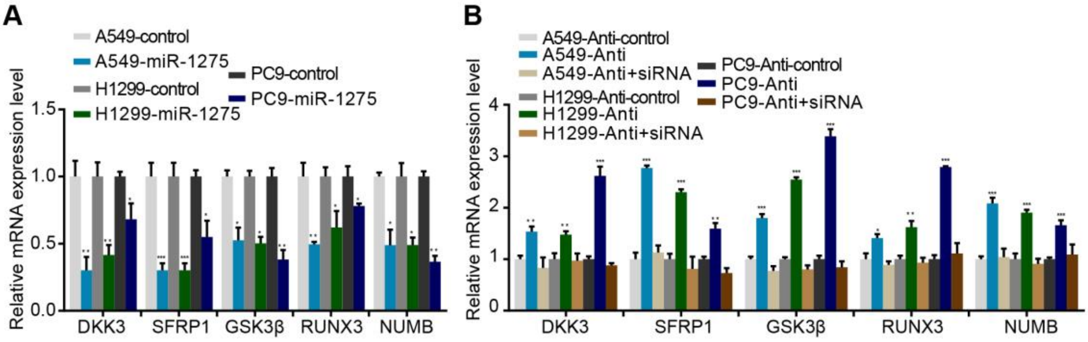

Figure S10: MiR-1275 targets Wnt/β-catenin and Notch signaling antagonists in vitro. (A) RT-qPCR analyses of DKK3, SFRP1, GSK3β, RUNX3, and NUMB expression levels in control cells and miR-1275-upregulated cells. (B) RT-qPCR analyses of DKK3, SFRP1, GSK3β, RUNX3, and NUMB expression levels in control cells, miR-1275 downregulated cells, and siRNA-treated cells. GAPDH was used as a loading control.

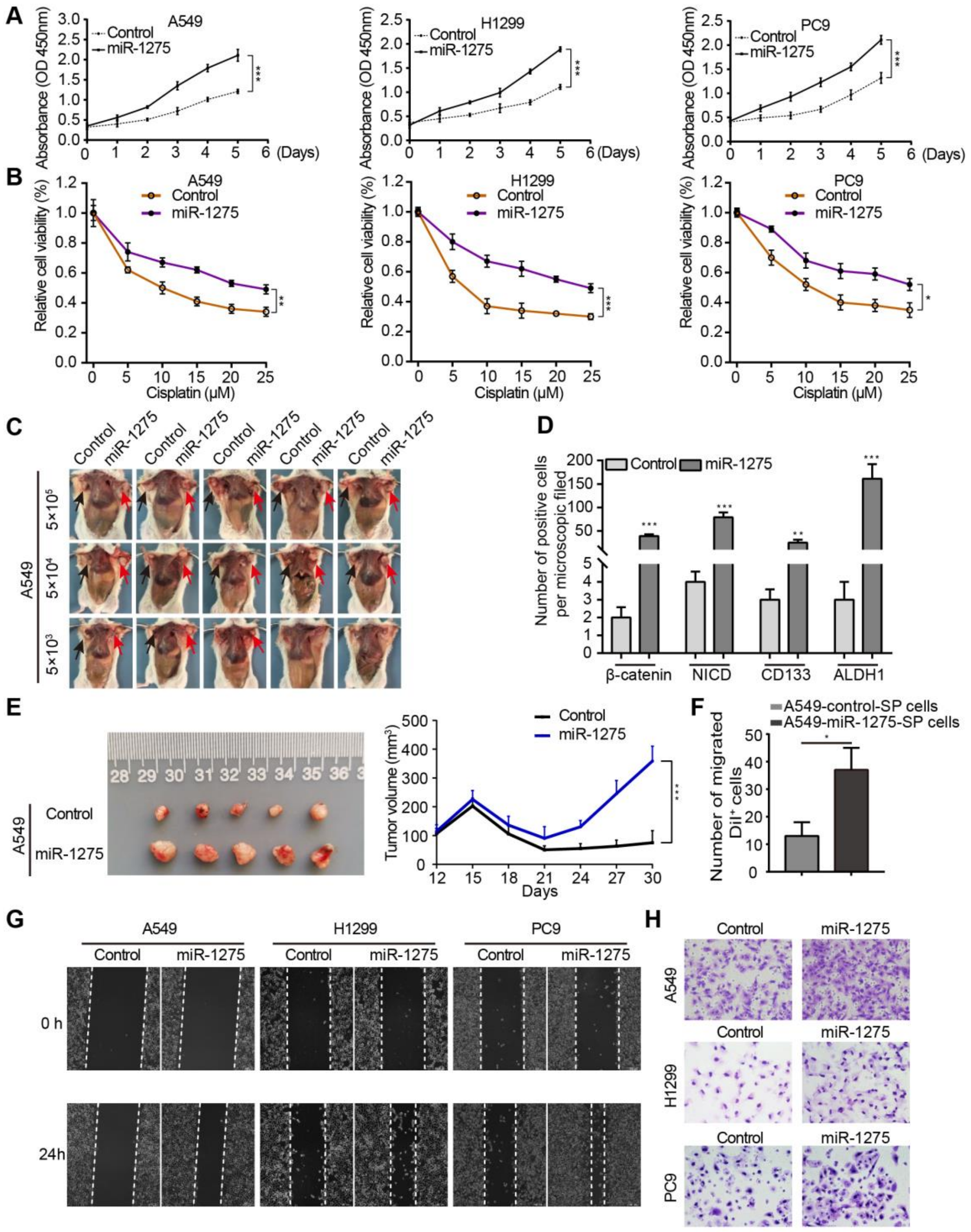

Figure S11: MiR-1275 promotes LUAD tumorigenicity, tumor recurrence and metastasis. (A) CCK8 assays showed the proliferation ability of the indicated cell lines. (B) Cell viability was analyzed using the CCK8 assay after treatment with CDDP. (C) Effects of miR-1275 on the tumorigenicity of A549 cells at different densities (5×10^5, 5×10^4, 5×10^3). Control groups are indicated with black arrows, and

the miR-1275-overexpressing groups are indicated with red arrows. Mice were sacrificed at 35 days. (D) Quantification data of β-catenin, NICD, CD133, and ALDH1. (E) Tumor growth in response to CDDP treatment. CDDP was intraperitoneally injected into mice at a concentration of 4 mg/kg every 2 days for a week. (F) Quantification of the total numbers of disseminated and metastatic cells in the primary tumor surroundings and the trunk regions of zebrafish. (G) Representative images of scratch assay in miR-1275-overexpressing cell lines and control cell lines. (H) Representative images of the Transwell invasion assay in miR-1275-overexpressing cell lines and control cell lines.

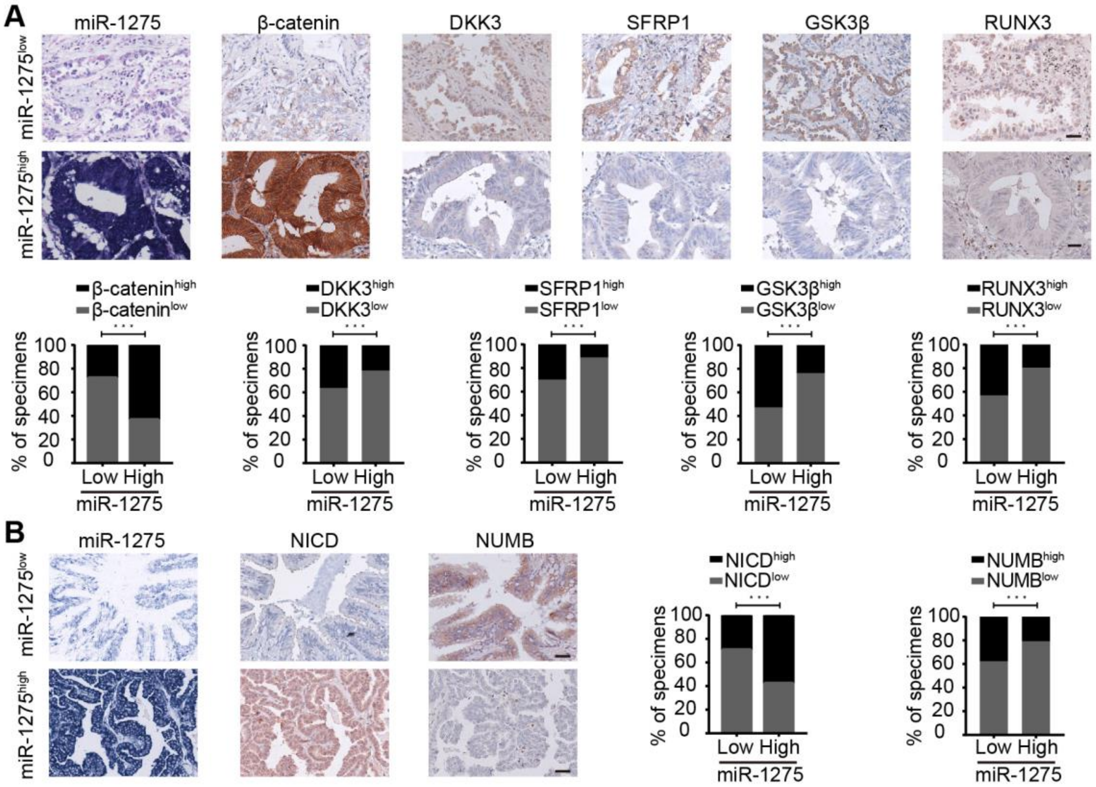

Figure S12: Clinical relevance of miR-1275 expression and its target genes in 558 LUAD specimens. (A) ISH images of miR-1275 and IHC images of β-catenin, DKK3, SFRP1, GSK3β, and RUNX3 in 558 LUAD tumor specimens. Percentage of samples showing high or low miR-1275 levels in relation to the expression levels of β-catenin, DKK3, SFRP1, GSK3β, and RUNX3 (chi-square test). (B) ISH images of miR-1275 and IHC images of NICD and NUMB in 558 LUAD tumor specimens. Percentage of samples showing high or low miR-1275 levels in relation to the expression levels of NICD and NUMB (chi-square test). Scale bar, 50 μm. *$P < 0.05$, **$P < 0.01$, ***$P < 0.001$.

Table S1: Clinicopathological characteristics of 5 pairs of patients in microarray analysis

Patients	Sex	Age (years)	Pathological type	Clinical stage	Mutations
Patient 1	Female	65	LUAD	IA (T1N0M0)	$EGFR^{+}KRAS^{-}ALK^{-}$
Patient 2	Female	60	LUAD	IIB (T1N1M0)	$EGFR^{-}KRAS^{-}ALK^{-}$
Patient 3	Male	54	LUAD	IIB (T3N0M0)	$EGFR^{-}KRAS^{-}ALK^{-}$
Patient 4	Male	57	LUAD	IIIA (T2N2M0)	$EGFR^{-}KRAS^{-}ALK^{-}$
Patient 5	Female	52	LUAD	IB (T2N0M0)	$EGFR^{-}KRAS^{+}ALK^{-}$

Table S3: Sequences of the miRNA primers used in the qPCR assay

miRNAs	Forward
miR-4516	GGGAGAAGGGTCGGGGCA
miR-4524b-5p	GCGATAGCAGCATAAGCCTGTCTC
miR-4657	CGCGAATGTGGAAGTGGTCTGAGGCAT
miR-3654	GACTGGACAAGCTGAGGAAA
miR-1275	GTGGGGGAGAGGCTGTCAA
miR-1298-5p	CGTTCATTCGGCTGTCCAGATGTA
miR-3915	CGCGCGCGTTGAGGAAAAGATGGTCTTATT
miR-1260a	GCGATCCCACCTCTGCCACCA

Table S4: Sequences of the mRNA primers used in the qPCR assay

Gene	5’primer	3’primer
DKK3	TGAGATGTTCCGCGAGGTTG	CAGGTTCACTTCTGATGATGCTTTA
GSK3β	GTTCTCGGTACTATAGGGCACCA	TCCTAGTAACAGCTCAGCCAACA
SFRP1	ACTGGCCCGAGATGCTTAAGTG	GAGATGTTCAATGATGGCCTCAGA
RUNX3	TCAACGACCTTCGCTTCGTG	ACCTTGATGGCTCGGTGGTA
NUMB	GCAGATGGACTCAGAGTTGTGGA	GTGCCATCACGGCATATGTAAGA
MYC	GGAGGCTATTCTGCCCATTTG	CGAGGTCATAGTTCCTGTTGGTG
WIF-1	CGAGGTACGCAATAGGAGTGTG	CACGTGCAGTTGTACTTGCAGTTAG
CD44	ACAAGCACAATCCAGGCAACTCC	TGGTGTTGTCCTTCCTTGCATTGG
MMP7	CATGATTGGCTTTGCGCGAG	GCATCTCCTCCGAGACCTGT
HIF-1α	AGCACAGTTACAGTATTCCAGCAGAC	TTAATTCATCAGTGGTGGCAGTGGTAG
VEGFA	GCCTTGCCTTGCTGCTCTACC	AGACATCCATGAACTTCACCACTTCG
CCND1	GTGCATCTACACCGACAACTCC	GTTCCACTTGAGCTTGTTCACC
HES1	GGACATTCTGGAAATGACAGTGA	AGCACACTTGGGTCTGTGCTC
HES3	ATGGAGAAAAAGCGCCGGGC	CACGCTCAACTCCAGGATGTCG
HEY1	CCGACGAGACCGAATCAATAAC	TCAGGTGATCCACAGTCATCTG
CCND3	ATGCGGAAGATGCTGGCTTACTG	AGACAGGTAGCGATCCAGGTAGTTC
SOX2	CCAAGATGCACAACTCGGAGA	CCGGTATTTATAATCCGGGTGCT
OCT4	GCTGGATGTCAGGGCTCTTTG	TTCAAGAGATTTATCGAGCACCTTC
ABCG2	TGCCCAGGACTCAATGCAAC	TCGATGCCCTGCTTTACCAAATA
CD133	AGTGGCATCGTGCAAACCTG	CTCCGAATCCATTCGACGATA
ALDH1	GCACGCCAGACTTACCTGTCCTA	GGCCTTCACTGCCTTGTCAAC
GAPDH	TGGGTGTGAACCATGAGAAGT	TGAGTCCTTCCACGATACCAA

Table S5: Correlation between miR-1275 expression and the clinicopathologic characteristics of 558 LUAD patients

**Terms**	**Cases**	**miR-1275 expression (high/low=279/279)**		
		**Low (%)**	**High (%)**	***P* value**
**Sex**				
Female	260	138 (53.1%)	122 (46.9%)	0.175
Male	298	141 (47.3%)	157 (52.7%)	
**Age (years)**				
<median (59)	272	139 (51.1%)	133 (48.9%)	0.611
≥median (59)	286	140 (49.0%)	146 (51.0%)	
**Primary tumor size**				
<2 cm	200	103 (51.5%)	97 (48.5%)	0.596
≥2 cm	358	176 (49.2%)	182 (50.8%)	
**Differentiation**				
Well/Moderate	487	265 (54.4%)	222 (45.6%)	< 0.001
Poor	71	14 (17.9%)	57 (80.3%)	
**T classification**				
Low (T1-T2)	496	254 (51.2%)	242 (48.8%)	0.106
High (T3-T4)	62	25 (40.3%)	37 (59.7%)	
**N classification**				
Low (N0-N1)	435	237 (54.5%)	198 (45.5%)	< 0.001
High (N2-N3)	123	42 (34.1%)	81 (65.9%)	
**Distant metastasis**				
NO	510	267 (52.4%)	243 (47.6%)	< 0.001
YES	48	12 (4.3%)	36 (12.9%)	
**Clinical stage**				
Low (Stages I-II)	412	234 (56.8%)	178 (43.2%)	< 0.001
High (Stages III-IV)	146	45 (30.8%)	101 (69.2%)	

Table S6: Correlation between miR-1275 expression and driver-gene status in the SYSUFH cohort

Genes	Mutation	Total cases	miR-1275 expression		
			**Low**	**High**	***P* value**
$EGFR^{mut}$	NO	72	32	40	0.185
	YES	74	41	33	
$KRAS^{mut}$	No	80	45	35	0.725
	YES	11	5	6	
ALK^{fus}	NO	62	35	27	0.512
	YES	6	2	4	

Pearson χ2 test or continuously corrected χ2 test. Only these patients have performed driver-gene detection previously. mut: mutation, fus: fusion.

Table S7: Univariate and multivariate Cox-regression analysis of the miR-1275 association with OS in 558 LUAD patients

	OS (Univariate analysis)		OS (Multivariate analysis)	
	**HR[a] (95% CI[b])**	***P* value**	**HR[a] (95% CI[b])**	***P* value**
**Sex**				
Female	1.00		1.00	
Male	1.31 (1.05-1.62)	0.015	0.83 (0.66-1.03)	0.092
**Age (years)**				
<median (59)	1.00		1.00	
≥median (59)	1.96 (1.57-2.451)	< 0.001	2.22 (1.76-2.78)	< 0.001
**Primary tumor size**				
<2 cm	1.00		1.00	
≥2 cm	1.45 (1.15-1.82)	0.001	1.14 (0.89-1.45)	0.312
**Differentiation**				
Well/Moderate	1.00		1.00	
Poor	1.63 (0.98-2.19)	< 0.001	1.39 (0.96-1.92)	0.039
**Clinical stage**				
Stage I	1.00		1.00	
Stage II	2.49 (1.78-3.48)	< 0.001	3.41 (2.13-5.47)	< 0.001
Stage III	6.95 (5.17-9.33)	< 0.001	12.41 (7.26-21.21)	< 0.001
Stage IV	25.19 (16.97-37.37)	< 0.001	31.60 (12.17-82.08)	< 0.001
**T classification**				
T1-T2	1.00		1.00	
T3-T4	4.30 (2.02-6.66)	0.012	1.12 (1.03-1.89)	0.130
**N classification**				
N0-N1	1.00		1.00	
N2-N3	3.52 (2.82-4.45)	< 0.001	1.31 (0.77-1.94)	0.030
**Distant metastasis**				
NO	1.00		1.00	
YES	10.03 (6.97-14.43)	< 0.001	1.60 (0.64-4.03)	0.016
**miR-1275 expression**				
miR-1275 low	1.00		1.00	
miR-1275 high	2.08 (1.67-2.58)	< 0.001	1.71 (1.36-2.17)	< 0.001

[a] Hazard ratio [b] Confidence interval

Table S8: Univariate and multivariate Cox-regression analysis of miR-1275 association with the OS of 327 LUAD patients from the SYSUFU clinical cohort

	OS (Univariate analysis)		OS (Multivariate analysis)	
	**HR[a] (95% CI[b])**	***P* value**	**HR[a] (95% CI[b])**	***P* value**
**Sex**				
Female	1.00		1.00	
Male	1.09 (0.82-1.44)	0.545	0.92 (0.68-1.25)	0.606
**Age (years)**				
<median (59)	1.00		1.00	
≥median (59)	2.21 (1.65-2.96)	< 0.001	2.49 (1.83-3.39)	< 0.001
**Primary tumor size**				
<2 cm	1.00		1.00	
≥2 cm	1.60 (1.11-2.31)	0.012	1.39 (0.94-2.05)	0.099
**Differentiation**				
Well/Moderate	1.00		1.00	
Poor	1.68 (0.82-1.95)	0.017	1.35 (0.60-2.35)	0.026
**Clinical stage**				
Stage I	1.00		1.00	
Stage II	2.42 (1.57-3.75)	< 0.001	3.25 (1.66-6.36)	0.001
Stage III	6.71 (4.57-9.87)	< 0.001	12.91 (6.25-26.64)	< 0.001
Stage IV	22.33 (13.09-38.11)	< 0.001	21.43 (13.63-30.83)	< 0.001
**T classification**				
T1-T2	1.00		1.00	
T3-T4	5.62 (4.95-7.98)	0.002	1.31 (1.088-6.11)	0.214
**N classification**				
N0-N1	1.00		1.00	
N2-N3	3.45 (2.62-4.37)	0.001	2.31 (1.45-3.68)	0.041
**Distant metastasis**				
NO	1.00		1.00	
YES	8.03 (4.86-13.29)	< 0.001	5.23 (3.01-8.34)	0.012
**miR-1275 expression**				
miR-1275 low	1.00		1.00	
miR-1275 high	2.45 (1.82-3.29)	< 0.001	2.10 (1.54-2.88)	< 0.001

[a] Hazard ratio [b] Confidence interval

Table S9: Univariate and multivariate Cox-regression analysis of miR-1275 association with the OS of 133 LUAD patients from the SYSUCC clinical cohort

	**OS (Univariate analysis)**		**OS (Multivariate analysis)**	
	**HR[a] (95% CI[b])**	***P* value**	**HR[a] (95% CI[b])**	***P* value**
**Sex**				
Female	1.00		1.00	
Male	1.44 (0.93-2.24)	0.102	1.29 (0.78-2.15)	0.319
**Age (years)**				
<median (59)	1.00		1.00	
≥median (59)	1.77 (1.13-2.77)	0.013	2.21 (1.34-3.66)	0.002
**Primary tumor size**				
<2 cm	1.00		1.00	
≥2 cm	1.21 (0.74-1.97)	0.451	0.78 (0.44-1.37)	0.382
**Differentiation**				
Well/Moderate	1.00		1.00	
Poor	1.63 (0.92-2.57)	0.013	1.38 (1.11-2.29)	0.014
**Clinical stage**				
Stage I	1.00		1.00	
Stage II	2.77 (1.33-5.79)	0.007	2.71 (1.42-8.61)	0.001
Stage III	8.96 (5.00-16.05)	< 0.001	5.99 (3.51-18.17)	0.001
Stage IV	36.93 (16.08-84.82)	< 0.001	7.34 (3.29-30.14)	0.003
**T classification**				
T1-T2	1.00		1.00	
T3-T4	4.31 (1.63-11.44)	0.003	1.34 (0.74-4.95)	0.067
**N classification**				
N0-N1	1.00		1.00	
N2-N3	3.04 (1.59-5.79)	0.001	1.81 (1.28-3.37)	0.002
**Distant metastasis**				
NO	1.00		1.00	
YES	16.34 (7.77-34.35)	< 0.001	61.26 (6.78-554.07)	< 0.001
**miR-1275 expression**				
miR-1275 low	1.00		1.00	
miR-1275 high	1.89 (1.21-2.97)	0.005	1.69 (1.12-2.67)	0.012

[a] Hazard ratio [b] Confidence interval

Table S10: Univariate and multivariate Cox-regression analysis of miR-1275 association with OS of 98 LUAD patients from the CHWH clinical cohort

	OS (Univariate analysis)		OS (Multivariate analysis)	
	**HR[a] (95% CI[b])**	***P* value**	**HR[a] (95% CI[b])**	***P* value**
**Sex**				
Female	1.00		1.00	
Male	2.61 (1.54-4.43)	< 0.001	2.46 (1.26-4.81)	0.009
**Age (years)**				
<median (59)	1.00		1.00	
≥median (59)	1.67 (0.94-2.60)	0.030	1.55 (0.85-2.23)	0.035
**Primary tumor size**				
<2 cm	1.00		1.00	
≥2 cm	1.89 (1.05-3.39)	0.034	1.24 (0.59-2.58)	0.572
**Differentiation**				
Well/Moderate	1.00		1.00	
Poor	0.46 (0.21-1.01)	0.053	3.64 (1.15-11.49)	0.027
**Clinical stage**				
Stage I	1.00		1.00	
Stage II	2.50(1.19-5.21)	0.014	2.63 (0.53-5.07)	0.036
Stage III	8.73 (4.37-18.24)	< 0.001	11.48 (2.55-51.72)	0.001
Stage IV	29.53 (12.35-70.59)	< 0.001	126.4 (5.31-310.7)	0.013
**T classification**				
T1-T2	1.00		1.00	
T3-T4	2.36 (1.79-4.44)	0.016	1.62 (0.71-2.04)	0.035
**N classification**				
N0-N1	1.00		1.00	
N2-N3	3.81 (1.76-8.25)	0.001	3.17 (1.92-7.02)	0.015
**Distant metastasis**				
NO	1.00		1.00	
YES	13.72 (6.36-22.56)	< 0.001	2.24 (1.01-6.29)	0.006
**miR-1275 expression**				
miR-1275 low	1.00		1.00	
miR-1275 high	3.14 (1.84-5.35)	< 0.001	2.07 (1.07-3.98)	0.030

[a] Hazard ratio [b] Confidence interval

Table S11: Correlation analysis between miR-1275 expression and the CTC count in 43 LUAD patients from the SYSUFU clinical cohort

**Terms**	**miR-1275 expression**		
	**Low (%)**	**High (%)**	***P* value**
**CTC counts**			
Negative < 1	13 (61.9%)	5 (22.7%)	0.009
Positive ⩾ 1	8 (38.1%)	17 (77.3%)	

Only 43 LUAD patients had previously performed CTC detection.

Table S12: Correlation between miR-1275 expression and the protein levels of β-catenin and NICD in 558 LUAD patients

**Terms**	**miR-1275 expression**		
	**Low (%)**	**High (%)**	***P* value**
**β-catenin expression**			
Low	201 (72.0%)	112 (40.1%)	
High	78 (28.0%)	167 (59.9%)	< 0.001
**NICD expression**			
Low	197 (70.6%)	118 (42.3%)	
High	82 (29.4%)	161 (57.7%)	< 0.001

Table S13: Correlation between miR-1275 expression and the protein levels of DKK3, SFRP1, GSK3β, RUNX3, and NUMB in 558 LUAD patients

Terms	miR-1275 expression		
	**Low (%)**	**High (%)**	***P* value**
**DKK3 expression**			
Low	178 (63.8%)	219 (78.5%)	
High	101 (36.2%)	60 (21.5%)	< 0.001
**SFRP1 expression**			
Low	196 (70.3%)	248 (88.9%)	
High	83 (29.7%)	31 (11.1%)	< 0.001
**GSK3β expression**			
Low	132 (47.3%)	213 (76.3%)	
High	147 (52.7%)	66 (23.7%)	< 0.001
**RUNX3 expression**			
Low	159 (57.0%)	224 (80.3%)	
High	120 (43.0%)	55 (19.7%)	< 0.001
**NUMB expression**			
Low	174 (62.4%)	221 (79.2%)	
High	105 (37.6%)	58 (20.8%)	< 0.001
